# Systematic review and meta-analysis of serious infections with tofacitinib and biologic disease-modifying antirheumatic drug treatment in rheumatoid arthritis clinical trials

**DOI:** 10.1186/s13075-015-0880-2

**Published:** 2015-12-15

**Authors:** Vibeke Strand, Sima Ahadieh, Jonathan French, Jamie Geier, Sriram Krishnaswami, Sujatha Menon, Tina Checchio, Thomas G. Tensfeldt, Elaine Hoffman, Richard Riese, Mary Boy, Juan J. Gómez-Reino

**Affiliations:** Biopharmaceutical Consultant, 306 Ramona Road, Portola Valley, CA 94028 USA; Pfizer Inc, Eastern Point Road, Groton, CT 06340 USA; Metrum Research Group, 2 Tunxis Rd Ste 112, Tariffville, CT 06081 USA; Pfizer Inc, 235 East 42nd Street, New York, NY 10017 USA; Complejo Hospitalario Universitario de Santiago de Compostela, Travesía de Choupana, s/n, 15706 Santiago de Compostela, Spain

**Keywords:** Infection, Meta-analysis, Rheumatoid arthritis, Tofacitinib, Disease-modifying antirheumatic drugs (biologic)

## Abstract

**Background:**

Tofacitinib is an oral Janus kinase inhibitor for the treatment of rheumatoid arthritis (RA). Tofacitinib modulates the signaling of cytokines that are integral to lymphocyte activation, proliferation, and function. Thus, tofacitinib therapy may result in suppression of multiple elements of the immune response. Serious infections have been reported in tofacitinib RA trials. However, limited head-to-head comparator data were available within the tofacitinib RA development program to directly compare rates of serious infections with tofacitinib relative to biologic agents, and specifically adalimumab (employed as an active control agent in two randomized controlled trials of tofacitinib).

**Methods:**

A systematic literature search of data from interventional randomized controlled trials and long-term extension studies with biologics in RA was carried out. Preferred Reporting Items for Systematic reviews and Meta-Analyses (PRISMA) consensus was followed for reporting results of the review and meta-analysis. Incidence rates (unique patients with events/100 patient-years) for each therapy were estimated based on data from randomized controlled trials and long-term extension studies using a random-effects model. Relative and absolute risk comparisons versus placebo used Mantel-Haenszel methods.

**Results:**

The search produced 657 hits. In total, 66 randomized controlled trials and 22 long-term extension studies met the selection criteria. Estimated incidence rates (95 % confidence intervals [CIs]) for abatacept, rituximab, tocilizumab, and tumor necrosis factor inhibitors were 3.04 (2.49, 3.72), 3.72 (2.99, 4.62), 5.45 (4.26, 6.96), and 4.90 (4.41, 5.44), respectively. Incidence rates (95 % CIs) for tofacitinib 5 and 10 mg twice daily (BID) in phase 3 trials were 3.02 (2.25, 4.05) and 3.00 (2.24, 4.02), respectively. Corresponding incidence rates in long-term extension studies were 2.50 (2.05, 3.04) and 3.19 (2.74, 3.72). The risk ratios (95 % CIs) versus placebo for tofacitinib 5 and 10 mg BID were 2.21 (0.60, 8.14) and 2.02 (0.56, 7.28), respectively. Risk differences (95 % CIs) versus placebo for tofacitinib 5 and 10 mg BID were 0.38 % (−0.24 %, 0.99 %) and 0.40 % (−0.22 %, 1.02 %), respectively.

**Conclusions:**

In interventional studies, the risk of serious infections with tofacitinib is comparable to published rates for biologic disease-modifying antirheumatic drugs in patients with moderate to severely active RA.

**Electronic supplementary material:**

The online version of this article (doi:10.1186/s13075-015-0880-2) contains supplementary material, which is available to authorized users.

## Background

Many disease-modifying antirheumatic drugs (DMARDs), particularly biologic agents, have become available with the approval of the first tumor necrosis factor inhibitors (TNFi). Despite documented efficacy in randomized controlled trials, serious infections (e.g., those requiring hospitalization and/or treatment with parenteral antibiotics) have been reported with varying degrees of risk and remain a major concern in patients treated with DMARDs [1]. Further, differences in trial designs pose challenges when analyzing the safety of DMARDs.

Several meta-analyses have compared the incidence of serious infections associated with various therapies for rheumatoid arthritis [1–7]. Many included trials of biologic DMARDs at dosages higher than those approved by regulatory authorities, or did not account for differences in duration of exposure or differences arising from pooling data from populations not restricted to moderate to severely active rheumatoid arthritis. Recently, Salgado et al. [2] included serious infection data for tofacitinib.

Tofacitinib is an oral Janus kinase inhibitor for the treatment of rheumatoid arthritis. Tofacitinib modulates the signaling of cytokines that are integral to lymphocyte activation, proliferation, and function [8, 9]. Thus, tofacitinib therapy may result in suppression of multiple elements of the immune response. The primary purpose of this analysis was to summarize and contextualize the risk of serious infections within the tofacitinib rheumatoid arthritis development program, based on a meta-analysis of randomized controlled trials and long-term extension studies of tofacitinib and biologic DMARDs. Adalimumab (with or without methotrexate [MTX]) was used as an active control agent in two randomized controlled trials of tofacitinib [10, 11]. However, patient numbers and exposure for adalimumab, and other control agents (placebo, with or without nonbiologic DMARDs), were limited due to trial design. Therefore, a meta-analytic approach was considered useful to overcome the inherent limitations of individual randomized controlled trials in the evaluation of safety outcomes.

## Methods

A systematic literature review identified safety data concerning serious infections from published interventional studies of biologic DMARDs and tofacitinib in patients with rheumatoid arthritis. Preferred Reporting Items for Systematic reviews and Meta-Analyses (PRISMA) consensus was followed for results reporting [12].

### Eligibility criteria

A search was conducted according to the Participants, Interventions, Comparisons, Outcome, Study Design (PICOS) statement, restricted to trials in moderate to severely active rheumatoid arthritis [12]. Interventions included tofacitinib and biologic agents currently licensed by the US Food and Drug Administration and/or European Medicines Agency for the treatment of rheumatoid arthritis. Comparisons were versus placebo (in trials in patients with inadequate response to DMARDs [DMARD-IR]) and MTX (for trials in MTX-naive patients). The endpoint of interest was serious infections (e.g., those requiring hospitalization and/or parenteral antibiotics [outcome]). Study designs included randomized controlled trials and long-term extension studies.

Data were selected to match the population of tofacitinib trials: adults with moderate to severely active rheumatoid arthritis. Trials were required to report data for serious infections and have sufficient data to calculate incidence rates (i.e., number of patients and patient-years of exposure). Studies that combined two biologic agents, duplicate reports, economic assessments, editorial comments, and case reports, were excluded.

### Information sources and search strategy

Articles published in English, in Medline, Embase, and Biosis, up to October 2013 were selected. Regulatory submissions within the US Food and Drug Administration Summary Basis for Approvals, as well as European Public Assessment Reports, were searched for serious infections data for biologic DMARDs approved for moderate to severely active rheumatoid arthritis.

Search criteria included keywords such as “rheumatoid arthritis” and clinical trials of DMARDs (abatacept, adalimumab, certolizumab, etanercept, golimumab, infliximab, rituximab, and tocilizumab) (see section 1 in Additional file 1). Citations within the assembled listing were cross-referenced for completeness, and articles were added based on the reverse search.

### Study selection

The initial search was performed by an information scientist with expertise in the field (C Hernandez). Independent reviewers screened articles by title and abstract, and any discrepancies were resolved by discussion (M Boy, R Riese, S Krishnaswami, V Strand). Subsequently, a complete reading of articles was performed, and articles that met the inclusion criteria were selected.

Tofacitinib data from five phase 2 randomized controlled trials (NCT00147498, NCT00413660, NCT00550446, NCT00603512, NCT00687193), six phase 3 randomized controlled trials (NCT00960440, NCT00847613, NCT00814307, NCT00856544, NCT00853385, NCT01039688), and two long-term extension studies (NCT00413699, NCT00661661) were extracted for analysis. Tofacitinib trials included patients who had failed nonbiologic DMARDs or biologic DMARDs, including TNFi, where the majority had failed MTX, and one trial in MTX-naive patients. For tofacitinib, only data from patients receiving 5 or 10 mg twice daily (BID) were included. Tofacitinib long-term extension studies were ongoing at the time of this analysis (August 2013 data cut); therefore, the database had not been locked (i.e., some values may change for the final, locked, study database). Publication details, demographics, treatments, and serious infections were collected. Trial quality was assessed using the Jadad scale [13].

### Summary measures

Three summary measures were assessed. The first estimated incidence rates (unique patients with events per 100 patient-years’ exposure) for each agent, utilizing a random-effects meta-analytic model [14] with a restricted maximum likelihood estimator for between-study variances. For tofacitinib, since there was access to patient-level data, incidence rates were obtained from pooling individual trials. For consistency, tofacitinib incidence rates were also estimated using the same meta-analysis model using summary incidence rates from each trial. The second and third strategies estimated relative risk (risk ratios) and risk differences for serious infections (with 95 % confidence intervals [CIs]), versus control, for each agent across randomized controlled trials up to rescue of patients randomized to receive placebo. Risk differences were expressed as differences in incidence percentage with 95 % CIs. Analysis was performed using the random-effects Mantel-Haenszel method [15].

### Synthesis of results

Study effects were plotted against the inverse of their standard errors to identify risk of publication bias, which was assessed visually using funnel plots for symmetry, and statistically, using the Egger test [16]. Heterogeneity across trials was assessed using the I^2^ statistic [17]. An I^2^ value >40 % indicated moderate to substantial heterogeneity [18]. When heterogeneity was present, possible causes were investigated via sensitivity analyses and meta-regression, using factors such as trial quality (Jadad scale) [13] and duration of exposure. *p* <0.05 was considered significant, except in the meta-regression analysis, where the significance level was 0.1. Additional sensitivity analyses, regardless of heterogeneity, excluded studies with zero incidence rates, long-term extension studies, studies with incidence rates reported on multiple occasions after different durations of exposure, and studies that included doses not approved for use by the US Food and Drug Administration or European Medicines Agency.

To explore additional clinical questions pertinent to use of these therapies in rheumatoid arthritis, namely as monotherapy or in MTX-naive patients, separate analyses were conducted for trials of monotherapy in DMARD-IR patients, in which DMARDs were discontinued prior to treatment initiation, and randomized controlled trials in MTX-naive patients. Incidence rates were calculated using the R (version 2.12.2) metafor package [19]. Risk ratios and risk differences were calculated using Review Manager Software (RevMan) version 5.2 [20]. However, as RevMan did not incorporate trials with zero incidence in both arms, a sensitivity analysis was performed using R.

## Results

### Trials included in the meta-analysis

**Fig. 1:**
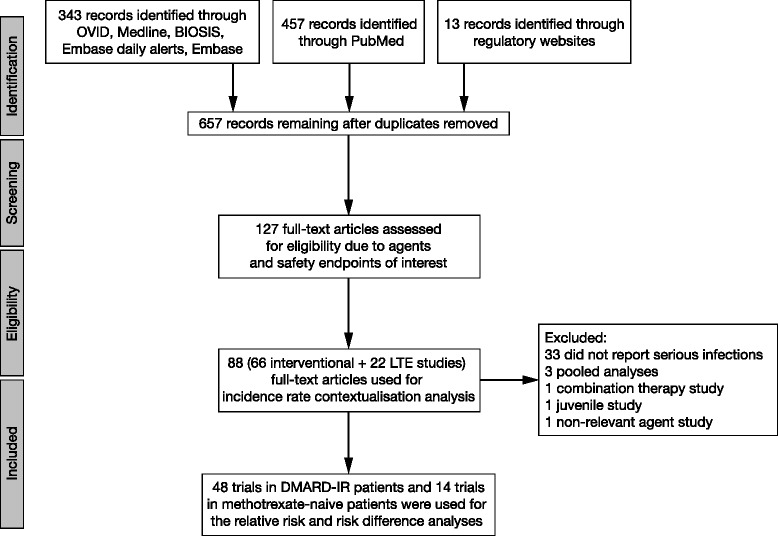
Flow diagram of the literature selection process. *DMARD*(*−IR*) disease-modifying antirheumatic drug(*−*inadequate responder), *LTE* long-term extension

Figure 1 illustrates the article selection process. The search identified 657 articles, of which 66 were randomized controlled trials and 22 were long-term extension studies, representing 40,512 patients. Data were extracted for analysis of serious infections as shown in Fig. 1, including 57, 11, 8, and 13 trials for TNFi, abatacept, rituximab, and tocilizumab, respectively. Tofacitinib results from phase 3 randomized controlled trials only, long-term extension studies only, and integrated data from pooled phase 2, phase 3, and long-term extension studies were included in the contextualization analysis. Individual trial characteristics for the 98 articles used in the analysis, including the 88 studies, are presented in section 2, Table 1 in Additional file 1.

A summary of age, gender, and trial duration by drug is presented in section 4 in Additional file 1. For relative risk and risk difference analyses there were 48 trials in DMARD-IR patients and 14 in MTX-naive patients. All trials included patients with moderate to severely active rheumatoid arthritis, with demographics and baseline characteristics comparable to those in the tofacitinib clinical development program. Median trial duration was longer for abatacept than for tofacitinib.

### Contextualization of serious infection rates for biologic DMARDs and tofacitinib

**Fig. 2:**
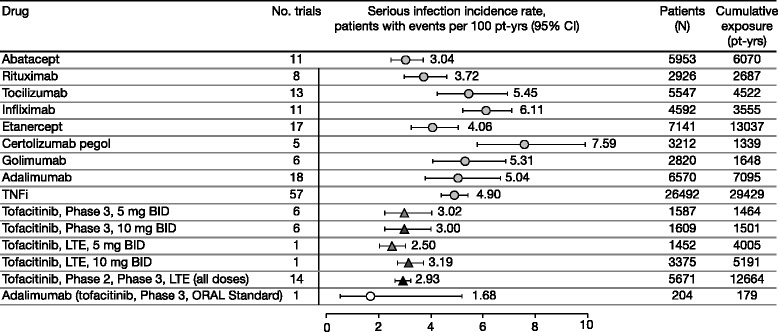
Incidence rates for serious infections with biologic DMARDs and tofacitinib across RCTs* and LTE studies*.* The results displayed did not include the continuity factor to account for zero incidence rates due to the low percentage of zero incidence rates for serious infections within these trials (<10 %). Tofacitinib data as of April 2013. *Clinical trial data published between 1999 and 2013. *BID* twice daily, *CI* confidence interval, *DMARD* disease-modifying antirheumatic drug, *LTE* long-term extension, *pt-yrs* patient-years, *RCT* randomized controlled trial, *TNFi* tumor necrosis factor inhibitors

Estimates of incidence rates (95 % CIs) for serious infections were: 3.04 (2.49, 3.72) for abatacept (I^2^ = 39.21 %, *p* = 0.083) with evidence of publication bias in the funnel plot (Egger test, *p* = 0.013); 3.72 (2.99, 4.62) for rituximab (I^2^ = 0 %, *p* = 0.68) without evidence of publication bias (*p* = 0.858); 5.45 (4.26, 6.96) for tocilizumab (I^2^ = 61.62 %, *p* = 0.0003) without evidence of publication bias (*p* = 0.178); and 4.90 (4.41, 5.44) across TNFi therapies (I^2^ = 64.56 %, *p* <0.0001) without evidence of publication bias (*p* = 0.113) (Fig. 2). Treatment duration and long-term, open-label studies were identified as causes of heterogeneity. For funnel plots, see section 6 in Additional file 1.

Exclusion of studies with Jadad score <3 (typically long-term, open-label studies), and including duration of exposure as a covariate, reduced I^2^ values significantly for therapies with high heterogeneity, including adalimumab (I^2^ = 25.3 %, *p* = 0.18), etanercept (I^2^ = 0 %, *p* = 0.151), and tocilizumab (I^2^ = 8.7 %, *p* = 0.085). Regardless of the analyses, incidence rate point estimates differed by <1 event/100 patient-years (adalimumab 5.04 vs. 4.29, etanercept 4.06 vs. 4.26, and tocilizumab 5.45 vs. 4.99).

Incidence rates (95 % CIs) for tofacitinib 5 and 10 mg BID in phase 3 randomized controlled trials were 3.02 (2.25, 4.05) and 3.00 (2.24, 4.02), respectively. Corresponding incidence rates in long-term extension studies were 2.50 (2.05, 3.04) and 3.19 (2.74, 3.72) (Fig. 2). The meta-analytic incidence rates (95 % CIs) for tofacitinib 5 and 10 mg BID were 2.83 (1.57, 5.08), and 2.31 (1.21, 4.41), respectively. There was no evidence of heterogeneity for tofacitinib 5 mg BID (I^2^ = 20.5 %, *p* = 0.532) or tofacitinib 10 mg BID (I^2^ = 24.1 %, *p* = 0.46), and no evidence of publication bias (Egger test *p* values: 0.288 and 0.354 for tofacitinib 5 and 10 mg, respectively). Sensitivity analyses were generally consistent with the primary analysis (see section 5 in Additional file 1).

### Risk ratio and risk difference for serious infection in patients with inadequate response to DMARDs

**Fig. 3:**
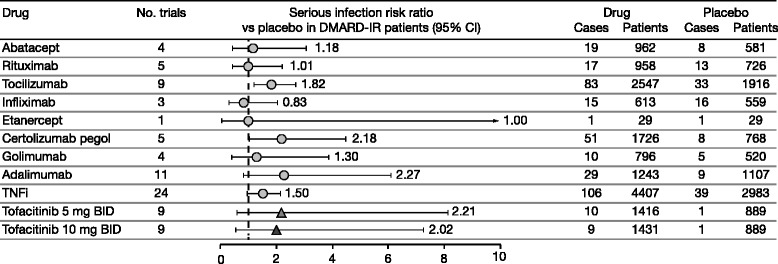
Risk ratios for biologic DMARDs and tofacitinib versus placebo across RCTs in DMARD-IR patients. Studies were predominantly on background methotrexate. *BID* twice daily, *CI* confidence interval, *DMARD*(*−IR*) disease-modifying antirheumatic drug(−inadequate responder), *RCT* randomized controlled trial, *TNFi* tumor necrosis factor inhibitors

**Fig. 4:**
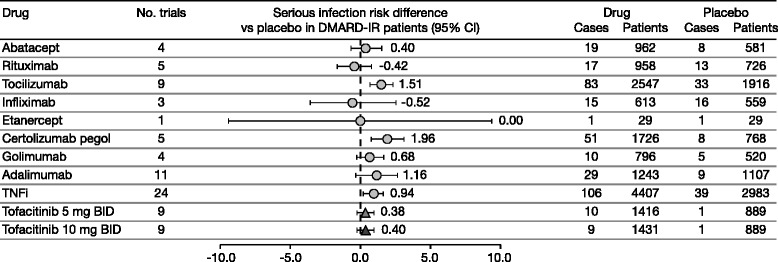
Risk differences for biologic DMARDs and tofacitinib versus placebo across RCTs in DMARD-IR patients. Studies were predominantly on background methotrexate. *BID* twice daily, *CI* confidence interval, *DMARD*(*−IR*) disease-modifying antirheumatic drug(−inadequate responder), *RCT* randomized controlled trial, *TNFi* tumor necrosis factor inhibitors

Comparative analyses of DMARD-IR trials (n = 48) are shown in Fig. 3 (relative risk) and Fig. 4 (risk difference). Studies were predominantly on background MTX. Relative risk values comparing DMARDs with placebo ranged from 0.83 to 2.27. There was only one randomized controlled trial with etanercept (DMARD-IR trials were generally not conducted in the late 1990s and early 2000s) [21], with a risk ratio (95 % CI) of 1.0 (0.07, 15.24) (Fig. 3). The risk ratio for TNFi versus placebo in DMARD-IR trials was 1.5 (1.00, 2.25). The risk ratio for tofacitinib versus placebo in phase 3 trials was 2.21 (0.60, 8.14) for 5 mg BID and 2.02 (0.56, 7.28) for 10 mg BID. There was no evidence of publication bias (Egger test *p* values: 0.17–0.94 across therapies).

Risk differences (95 % CI) versus placebo were 0.94 % (0.25 %, 1.63 %) for TNFi therapies, 0.4 % (−0.72 %, 1.51 %) for abatacept, 1.51 % (0.68 %, 2.33 %) for tocilizumab, −0.42 % (−1.63 %, 0.79 %) for rituximab, 0.38 % (−0.24 %, 0.99 %) for tofacitinib 5 mg BID, and 0.40 % (−0.22 %, 1.02 %) for tofacitinib 10 mg BID (Fig. 4). With the exception of adalimumab (*p* = 0.04) there was no evidence of publication bias (Egger test *p* values: 0.27–0.86 across other therapies).

In relative risk and risk differences analyses, I^2^ values were <40 % in all cases with one exception: the I^2^ value for the adalimumab risk difference analysis was 49 %, which reduced to 15 % following exclusion of a study with the highest exposure duration (approximately 9 months).

### Serious infection risk in monotherapy and methotrexate-naive populations

**Fig. 5:**
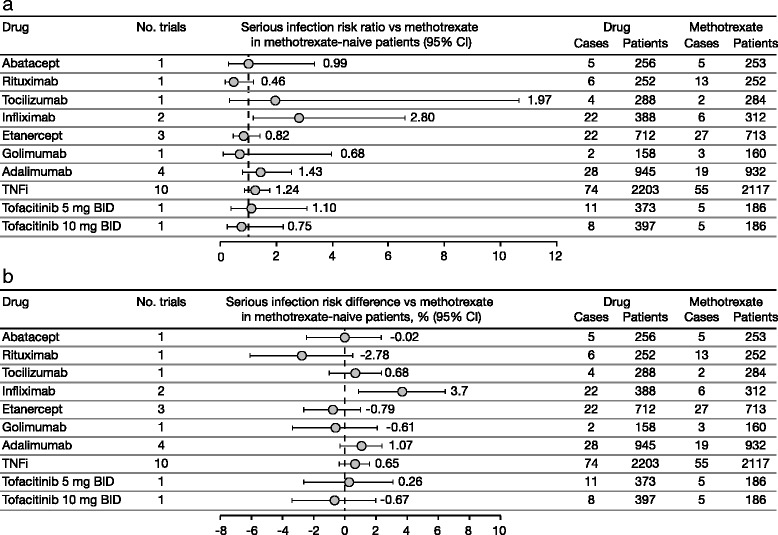
Risk ratios (**a**) and risk differences (**b**) for serious infections across RCTs in methotrexate-naive patients. Tofacitinib data are from the phase 3 ORAL Start study. *BID* twice daily, *CI* confidence interval, *RCT* randomized controlled trial, *TNFi* tumor necrosis factor inhibitors

Our database included five randomized controlled trials of TNFi monotherapy in DMARD-IR patients and 14 randomized controlled trials of biologic DMARDs in MTX-naive patients. Among monotherapy trials, the incidence rate (95 % CI) estimate was 5.34 (1.75, 16.3) events/100 patient-years (I^2^ = 39.92, *p* = 0.23) with borderline evidence of publication bias (*p* = 0.074) (see Figure S9 in Additional file 1). Within the MTX-naive population, risk ratio (95 % CI) point estimates versus MTX were 0.46–2.8 across biologic DMARDs, 1.24 (0.87, 1.77) for TNFi, 1.10 (0.39, 3.11) for tofacitinib 5 mg BID monotherapy, and 0.75 (0.25, 2.26) for tofacitinib 10 mg BID monotherapy (see sections 3 and 5.2.2 in Additional file 1). Risk difference point estimates versus MTX ranged from −2.78 % to 3.70 % across biologic DMARDs, 0.65 % (−0.33 %, 1.63 %) for TNFi, 0.26 % (−2.63 %, 3.15 %) for tofacitinib 5 mg BID monotherapy, and −0.67 % (−3.38, 2.03) for tofacitinib 10 mg BID (Fig. 5, and sections 3 and 5.3.2 in Additional file 1).

## Discussion

These analyses, which included data from 66 randomized controlled trials and 22 long-term extension studies, provide a substantial integrated assessment of serious infection risk in patients with moderate to severely active rheumatoid arthritis, and evaluate results from the tofacitinib clinical development program in the context of TNFi and other biologic DMARDs. As identified in a commentary on a recent meta-analysis of serious infections data 106 trials of biologic DMARDs in rheumatoid arthritis [7], selection of an appropriate treatment for a given patient is informed by how infection incidence vary according to drug, and individual patient characteristics [22]. Thus, an advantage of the analytical approach used here is that estimates of serious infection risk were obtained for individual DMARDs, with a further breakdown of risk according to prior treatment (MTX-naive versus DMARD inadequate responders). Despite limitations inherent in the design of randomized controlled trials (including small sample sizes, limited trial durations, and unequal exposure between active and control [placebo] therapies for ethical reasons), the typical onset of serious infections observed (within the first 3–12 months of treatment) and inclusion of data from long-term extension studies provide a holistic view of serious infection risk in randomized controlled trials.

We attempted to estimate incidence rates as precisely as possible, using all available data for each therapy, and provided comparative (relative risk) and absolute (risk difference) assessments versus controls, using data from randomized controlled trials only. Collectively, the incidence rates, risk ratio, and risk difference estimates for approved doses of biologic DMARDs do not indicate any significant differences in the risk of serious infections with tofacitinib compared with TNFi and biologic DMARDs in DMARD-IR patients. Numerical differences were noted, e.g., between tofacitinib and tocilizumab, when incidence rates were compared, but risk ratio and risk difference estimates indicated similar risk. CIs for risk differences appeared more precise compared with risk ratios, likely due to the limited number of events in the placebo groups over 3 months’ exposure (one event across all tofacitinib randomized controlled trials).

The present study explored two additional clinical questions pertinent to the clinical use of these therapies in rheumatoid arthritis, namely as monotherapy or compared with MTX in MTX-naive patients. The incidence rate (95 % CI) for serious infection across TNFi (5.34 [1.75, 16.3]) was consistent with the incidence rate from DMARD-IR studies (4.9 [4.4, 5.4]). In two tofacitinib monotherapy trials of 6 months’ duration (in DMARD-IR patients) [10, 23], no serious infections were reported with tofacitinib 5 mg BID (n = 292) or placebo (n = 181, 3-month period) and one event with tofacitinib 10 mg BID (n = 306). Additional data are needed to ascertain whether tofacitinib is associated with a lower risk of serious infections when administered as monotherapy versus combination therapy (with MTX). In contrast, studies in MTX-naive patients provide a robust comparison to MTX as they allow comparison to active control over the full study duration. While CIs from the relative risk and risk difference analyses in this population include the null, point estimates indicate a similar magnitude of risk of serious infections with biologic DMARDs in MTX-naive patients (see section 3 in Additional file 1). An analysis of tofacitinib in biologic DMARD-naive patients versus biologic DMARD-IR patients with rheumatoid arthritis, conducted separately and not as part of this meta-analytic framework, showed that the incidence rates for events of special interest, including serious infections, appear consistent between the two populations [24].

Two tofacitinib studies provide important direct evidence to assess the risk of serious infections [11, 25]. ORAL Start, a 2-year study comparing tofacitinib with MTX in MTX-naive patients [25], showed a similar incidence of serious infections with tofacitinib 5 mg BID (11/373 [incidence rate (95 % CI) 1.82 (1.01, 3.28)]) and tofacitinib 10 mg BID (8/397 [incidence rate (95 % CI) 1.23 (0.62, 2.46)]) compared with MTX (5/186 [incidence rate (95 % CI) 1.87 (0.78, 4.49)]). ORAL Standard [11] demonstrated a higher incidence of serious infections with tofacitinib 5 (7/204 [3.4 %]) and 10 mg BID (8/201 [4.0 %]) (both plus MTX) versus adalimumab plus MTX (3/204 [1.5 %]). However, the incidence rate (95 % CI) for serious adverse events with adalimumab plus MTX from this 1-year trial (1.68/100 patient-years [0.54, 5.02]) was in the lower end of the range of published adalimumab studies (range: 0.7 to 37 events/100 patient-years) and lower than the incidence rate estimate from this entire analysis (5.04 events/100 patient-years [3.8, 6.69]). Patient demographics and disease characteristics in the ORAL Standard study may not explain this as they do not present unique differences relative to other reported adalimumab studies.

Limitations of this analysis include the reliance upon published data describing the use of biologic agents in patients with rheumatoid arthritis. While publication bias typically results in an attenuation of effect estimates for safety endpoints, resultant bias is not measureable. Second, study-specific covariates (i.e., baseline comorbidities, stage and duration of disease, variations by geographic region and publication year) were not evaluated. Consequently, the incidence rate data presented may not be directly applicable to all patient subgroups with rheumatoid arthritis. Additionally, the trials represent a range with respect to patient-years of exposure and length of controlled treatment (longer- versus shorter-term). If the risk of serious infection varies over time, then inclusion of shorter-term trials could have biased results. A weak relationship between incidence rates and study duration can be noted, with higher incidence rates for shorter study durations. This could be related to the phenomenon of “depletion of susceptibles, survival bias, or higher disease activity at baseline” [26, 27]. Finally, patient dropout must be considered as a possible influence on the results [28]. For trials that reported incidence rates, it is reasonable to expect that individual patient dropout information would be factored into the exposure duration. However, for trials that did not directly report incidence rates, nominal exposure time was used to impute the incidence rate. This method has the potential to underestimate the true incidence rate as well as the variance estimate because it assumes no dropouts. For tofacitinib, the availability of patient-level data allowed incidence rates to be calculated based on individual exposure information. The pooled and meta-analytic incidence rates were comparable.

A network meta-analysis was not pursued because of differences in randomized controlled trials conducted in RA with respect to patient populations and comparator therapy, e.g., MTX in MTX-naïve patients, placebo + background DMARD therapy in DMARD-IR patients, and placebo in monotherapy trials. Such an analysis would have required an assumption to be made that the control arms in these settings are the same in order to have a node that could serve to compare incidence rates between therapies. This was an assumption was not supported by the evidence in the literature collected over the past decade and would have increased the potential for combining studies that were not appropriately similar to allow quantitative comparisons between biologic DMARDs and tofacitinib to be generated. This was also the reason why MTX-naïve and DMARD-IR comparisons were performed separately, more so since the duration of placebo treatment in DMARD-IR studies is generally much shorter (3–6 months) than the duration of MTX treatment in MTX-naïve patients (1–2 years), and this analysis shows differences in the incidence of infections between the different populations. While a network meta-analysis could be performed for each population separately, it was considered unlikely to provide additional value beyond the pairwise approach used in this analysis to qualitatively compare the therapies.

The results of the present analysis corroborate findings from other recent analyses [1, 3, 4]. Leombruno et al. reported an odds ratio (95 % CI) of 1.21 (0.89, 1.63) for serious infection with TNFi administered at recommended doses [4]. Thompson and colleagues evaluated studies among DMARD-naive patients with early rheumatoid arthritis, treated with TNFi, and found the odds ratio for serious infection (TNFi versus MTX control) was not statistically more frequent (1.28 [0.82, 2.00]) [3]. Michaud et al. also reported a higher risk of serious infection with adalimumab, certolizumab pegol, and infliximab, which appears to contribute to higher rates of discontinuations [1]. Within our analysis, the risk ratio (95 % CI) for TNFi was 1.45 (0.98, 2.17) and 1.25 (0.86, 1.81) in the DMARD-IR and MTX-naive populations, respectively. Similarly, Salgado et al. reported that the risk ratio (95 % CI) for serious infections was not significantly elevated in patients receiving protein kinase inhibitors compared with controls (fostamatinib: 1.07 [0.40, 2.91]; Janus kinase inhibitors: 1.68 [0.71, 3.91]) [2]. In this study, the reported risk ratio (95 % CI) for serious infection in patients exposed to tofacitinib in randomized controlled trials (across multiple indications) was 1.57 (0.65, 3.82), in line with the present analysis.

Concomitant corticosteroid intake has a definitive role in the risk of serious infections. However, the detailed information necessary to allow such analysis in a meta-analysis framework was not reported in most of the articles – thus precluding inclusion in this analysis. Concomitant corticosteroid intake was investigated for tofacitinib as part of a multivariate Cox proportional hazards analysis of risk factors. The results confirm that, as with biologic DMARDs, concomitant corticosteroid intake results in increase in the risk of serious infections [29]. Further, a thorough evaluation of serious infection risk should be contextualized within treatment benefits.

## Conclusions

The results of this meta-analysis indicate that in interventional studies, the rates of serious infections associated with tofacitinib in patients with moderate to severely active rheumatoid arthritis are within the range of those reported for biologic DMARDs.

## Additional file

Additional file 1:
**Details of literature search strategy; table of characteristics of individual studies included in the meta-analysis; summary table of risk ratios and risk differences across trials in methotrexate-naive patients; summary table of age, gender and study duration by drug; tables of incidence rates and risk ratios determined in sensitivity analysis; forest plots of incidence rates for serious infections by drug; forest plots of risk ratios and risk differences for serious infections by drug; funnel plots for evaluation of potential publication bias.** (DOC 5007 kb)

## Abbreviations

BID: Twice daily
CI: Confidence interval
DMARD(−IR): Disease-modifying antirheumatic drug(−inadequate responder)
MTX: Methotrexate
TNFi: Tumor necrosis factor inhibitors
