# Supplementary material for: Systematic review and meta-analysis of serious infections with tofacitinib and biologic disease-modifying antirheumatic drug treatment in rheumatoid arthritis clinical trials
Source: Arthritis Res Ther. 2015 Dec 15;17:362. doi: 10.1186/s13075-015-0880-2 (PMC4704538; doi:10.1186/s13075-015-0880-2)
Supplement: Additional file 1: — Details of literature search strategy; table of characteristics of individual studies included in the meta-analysis; summary table of risk ratios and risk differences across trials in methotrexate-naive patients; summary table of age, gender and study duration by drug; tables of incidence rates and risk ratios determined in sensitivity analysis; forest plots of incidence rates for serious infections by drug; forest plots of risk ratios and risk differences for serious infections by drug; funnel plots for evaluation of potential publication bias. (DOC 5007 kb) [file 13075_2015_880_MOESM1_ESM.doc]

**ADDITIONAL material**

**Systematic review and meta-analysis of serious infections with tofacitinib and biologic disease-modifying antirheumatic drug treatment in rheumatoid arthritis clinical trials**

Vibeke Strand,^1^ Sima Ahadieh,^2^ Jonathan French,^3^ Jamie Geier,^4^ Sriram Krishnaswami,^2^ Sujatha Menon,^2^ Tina Checchio,^2^ Thomas G Tensfeldt,^2^ Elaine Hoffman,^2^ Richard Riese,^2^ Mary Boy,^2^ Juan J Gómez-Reino^5*^

*^1^Biopharmaceutical Consultant, Portola Valley, California, USA; ^2^Pfizer Inc, Groton, Connecticut, USA; ^3^Metrum Research Group, Tariffville, Connecticut, USA; ^4^Pfizer Inc, New York, New York, USA; ^5^Complejo Hospitalario Universitario de Santiago de Compostela, Santiago de Compostela, Spain*

*Corresponding author

TABLE OF CONTENTS

[LIST OF TABLES 3](#__RefHeading___Toc411864006)

[LIST OF FIGURES 3](#__RefHeading___Toc411864007)

[1. search strategy 6](#__RefHeading___Toc411864008)

[2. INDIVIDUAL TRIAL CHARACTERISTICS FOR ARTICLES USED IN THE
meta-ANALYSIS 9](#__RefHeading___Toc411864009)

[3. Summary of risk ratio and risk difference results across biologic dmards and tofacitinib in methotrexate naIve trials 16](#__RefHeading___Toc411864010)

[4. summary of age, gender and study duration by drug 18](#__RefHeading___Toc411864011)

[5. Sensitivity Analyses TABLES 19](#__RefHeading___Toc411864012)

[5.1. Incidence Rate 19](#__RefHeading___Toc411864013)

[5.2. Risk Ratio by Drug 30](#__RefHeading___Toc411864014)

[5.2.1. DMARD-IR Population 30](#__RefHeading___Toc411864015)

[5.2.2. MTX-naive Population 38](#__RefHeading___Toc411864016)

[5.3. Risk Difference by Drug 43](#__RefHeading___Toc411864017)

[5.3.1. DMARD-IR Population 43](#__RefHeading___Toc411864018)

[5.3.2. MTX-naive Population 51](#__RefHeading___Toc411864019)

[6. FUNNEL PLOTS 56](#__RefHeading___Toc411864020)

LIST OF TABLES

[Table 1. Trials of biologic disease-modifying antirheumatic drugs and tofacitinib in patients with moderate to severely active rheumatoid arthritis who were included in the serious infections analyses 9](#__RefHeading___Toc411863909)

[Table 2. Risk ratios and risk differences for rates of serious infection events associated with approved biologic DMARDs and tofacitinib in randomized clinical trials conducted in methotrexate-naive patients 16](#__RefHeading___Toc411863910)

[Table 3. Summary of age, gender and study duration by drug 18](#__RefHeading___Toc411863911)

[Table 4. Sensitivity analysis for contextualization: IR (95% CI) [N] 19](#__RefHeading___Toc411863912)

[Table 5. Sensitivity analysis using R 2.12.2 for trials with zero events. RR for approved biologics vs placebo in DMARD-IR population 20](#__RefHeading___Toc411863913)

LIST OF FIGURES

[Figure 1. Abatacept Incidence Rate 21](#__RefHeading___Toc406748727)

[Figure 2. Rituximab Incidence Rate 22](#__RefHeading___Toc406748728)

[Figure 3. Tocilizumab Incidence Rate 23](#__RefHeading___Toc406748729)

[Figure 4. Infliximab Incidence Rate 24](#__RefHeading___Toc406748730)

[Figure 5. Etanercept Incidence Rate 25](#__RefHeading___Toc406748731)

[Figure 6. Certolizumab Pegol Incidence Rate 26](#__RefHeading___Toc406748732)

[Figure 7. Golimumab Incidence Rate 27](#__RefHeading___Toc406748733)

[Figure 8. Adalimumab Incidence Rate 28](#__RefHeading___Toc406748734)

[Figure 9. Incidence Rates of Adalimumab and Certolizumab from Monotherapy Trials in DMARD-IR Population 29](#__RefHeading___Toc406748735)

[Figure 10. Abatacept Risk Ratio (DMARD-IR population) 30](#__RefHeading___Toc406748736)

[Figure 11. Rituximab Risk Ratio (DMARD-IR population) 31](#__RefHeading___Toc406748737)

[Figure 12. Tocilizumab Risk Ratio (DMARD-IR population) 32](#__RefHeading___Toc406748738)

[Figure 13. Infliximab Risk Ratio (DMARD-IR population) 32](#__RefHeading___Toc406748739)

[Figure 14. Golimumab Risk Ratio (DMARD-IR population) 33](#__RefHeading___Toc406748740)

[Figure 15. Certolizumab Risk Ratio (DMARD-IR population) 33](#__RefHeading___Toc406748741)

[Figure 16. Adalimumab Risk Ratio (DMARD-IR population) 34](#__RefHeading___Toc406748742)

[Figure 17. Etanercept Risk Ratio (DMARD-IR population) 35](#__RefHeading___Toc406748743)

[Figure 18. Tofacitinib 5 mg BID Risk Ratio (DMARD-IR population) 35](#__RefHeading___Toc406748744)

[Figure 19. Tofacitinib 10 mg BID Risk Ratio (DMARD-IR population) 36](#__RefHeading___Toc406748745)

[Figure 20. TNF Inhibitors Risk Ratio (DMARD-IR population) 37](#__RefHeading___Toc406748746)

[Figure 21. Abatacept Risk Ratio (MTX-naive population) 38](#__RefHeading___Toc406748747)

[Figure 22. Rituximab Risk Ratio (MTX-naive population) 38](#__RefHeading___Toc406748748)

[Figure 23. Infliximab Risk Ratio (MTX-naive population) 39](#__RefHeading___Toc406748749)

[Figure 24. Golimumab Risk Ratio (MTX-naive population) 39](#__RefHeading___Toc406748750)

[Figure 25. Adalimumab Risk Ratio (MTX-naive population) 40](#__RefHeading___Toc406748751)

[Figure 26. Etanercept Risk Ratio (MTX-naive population) 40](#__RefHeading___Toc406748752)

[Figure 27. Tofacitinib 5 mg BID Risk Ratio (MTX-naive population) 41](#__RefHeading___Toc406748753)

[Figure 28. Tofacitinib 10 mg BID Risk Ratio (MTX-naive population) 41](#__RefHeading___Toc406748754)

[Figure 29. Tocilizumab Risk Ratio (MTX-naive population) 41](#__RefHeading___Toc406748755)

[Figure 30. TNF Inhibitors Risk Ratio (MTX-naive population) 42](#__RefHeading___Toc406748756)

[Figure 31. Abatacept Risk Difference (DMARD-IR population) 43](#__RefHeading___Toc406748757)

[Figure 32. Rituximab Risk Difference (DMARD-IR population) 43](#__RefHeading___Toc406748758)

[Figure 33. Tocilizumab Risk Difference (DMARD-IR population) 44](#__RefHeading___Toc406748759)

[Figure 34. Infliximab Risk Difference (DMARD-IR population) 45](#__RefHeading___Toc406748760)

[Figure 35. Golimumab Risk Difference (DMARD-IR population) 45](#__RefHeading___Toc406748761)

[Figure 36. Certolizumab Risk Difference (DMARD-IR population) 46](#__RefHeading___Toc406748762)

[Figure 37. Adalimumab Risk Difference (DMARD-IR population) 47](#__RefHeading___Toc406748763)

[Figure 38. Etanercept Risk Difference (DMARD-IR population) 47](#__RefHeading___Toc406748764)

[Figure 39. Tofacitinib 5 mg BID Risk Difference (DMARD-IR population) 48](#__RefHeading___Toc406748765)

[Figure 40. Tofacitinib 10 mg BID Risk Difference (DMARD-IR population) 49](#__RefHeading___Toc406748766)

[Figure 41. TNF Inhibitors Risk Difference (DMARD-IR population) 50](#__RefHeading___Toc406748767)

[Figure 42. Abatacept Risk Difference (MTX-naive population) 51](#__RefHeading___Toc406748768)

[Figure 43. Rituximab Risk Difference (MTX-naive population) 51](#__RefHeading___Toc406748769)

[Figure 44. Infliximab Risk Difference (MTX-naive population) 52](#__RefHeading___Toc406748770)

[Figure 45. Golimumab Risk Difference (MTX-naive population) 52](#__RefHeading___Toc406748771)

[Figure 46. Adalimumab Risk Difference (MTX-naive population) 53](#__RefHeading___Toc406748772)

[Figure 47. Etanercept Risk Difference (MTX-naive population) 53](#__RefHeading___Toc406748773)

[Figure 48. Tofacitinib 5 mg BID Risk Difference (MTX-naive population) 54](#__RefHeading___Toc406748774)

[Figure 49. Tofacitinib 10 mg BID Risk Difference (MTX-naive population) 54](#__RefHeading___Toc406748775)

[Figure 50. Tocilizumab Risk Difference (MTX-naive population) 54](#__RefHeading___Toc406748776)

[Figure 51. TNF Inhibitors Risk Difference (MTX-naive population) 55](#__RefHeading___Toc406748777)

[Figure 52. Funnel plot for Abatacept Incidence Rate 56](#__RefHeading___Toc406748778)

[Figure 53. Funnel plot for Rituximab Incidence Rate 57](#__RefHeading___Toc406748779)

[Figure 54. Funnel plot for Tocilizumab Incidence Rate 58](#__RefHeading___Toc406748780)

[Figure 55. Funnel plot for Infliximab Incidence Rate 59](#__RefHeading___Toc406748781)

[Figure 56. Funnel plot for Etanercept Incidence Rate 60](#__RefHeading___Toc406748782)

[Figure 57. Funnel plot for Certolizumab Pegol Incidence Rate 61](#__RefHeading___Toc406748783)

[Figure 58. Funnel plot for Golimumab Incidence Rate 62](#__RefHeading___Toc406748784)

[Figure 59. Funnel plot for Adalimumab Incidence Rate 63](#__RefHeading___Toc406748785)

[Figure 60. Funnel plot for TNF Inhibitors Incidence Rate 64](#__RefHeading___Toc406748786)

# search strategy

Articles published in English, in Medline, Embase and Biosis through October 2013, were selected. Additionally, regulatory submission documents (ie clinical and statistical reviews) within the US FDA Summary Basis for Approvals (SBAs) as well as European Public Assessment Reports (EPARs) were searched for information on serious infections for biologic therapies approved for the treatment of moderate to severe RA.

Search Strategy:

--------------------------------------------------------------------------------

1 *rheumatoid arthritis/ (140707)

2 rheumatoid arthritis.ti. (131703)

3 *Arthritis, Rheumatoid/ (139921)

4 ra.ti. (9888)

5 1 or 2 or 3 or 4 (191623)

6 (cost$ or economic$).ti. (282056)

7 (etanercept or enbrel or tunex).ti,ab. or *etanercept/ or *enbrel/ or *tunex/ (11103)

8 (adalimumab or humira or trudexa).ti,ab. or *adalimumab/ or *humira/ or *trudexa/ (7231)

9 (methotrexate or mtx).ti,ab. or *methotrexate/ or *mtx/ (114558)

10 (abatacept or orencia).ti,ab. or *abatacept/ or *orencia/ (1608)

11 (infliximab or centnf or remicade or avakine).ti,ab. or *infliximab/ or *centnf/ or *remicade/ or *avakine/ (19638)

12 (atlizumab or tocilizumab or actemra or roactemra).ti,ab. or *atlizumab/ or *tocilizumab/ or *actemra/ or *roactemra/ (1465)

13 (leflunomide or arava or repso).ti,ab. or *leflunomide/ or *arava/ or *repso/ (4783)

14 (sulfasalazine or sulphasalazine or salazosulfapyridine or azulfidine or salazopyrin or salazopyrine).ti,ab. or *salazosulfapyridine/ or *sulfasalazine/ or *sulphasalazine/ or *azulfidine/ or *salazopyrin/ or *salazopyrine/ (14358)

15 (hydroxychloroquine or chloroquine).ti,ab. or *hydroxychloroquine/ or *chloroquine/ (48646)

16 (certolizumab or cimzia or cimziat).ti,ab. or *certolizumab/ or *cimzia/ or *cimziat/ (1120)

17 (golimumab or simponi).ti,ab. or *golimumab/ or *simponi/ (575)

18 (adverse event$ or ae or aes or adverse reaction$ or SAE or saes or "side effect" or "side effects").ti,ab. (705380)

19 (mortality or (serious adj2 infection$) or herpes zoster or malignant or malignancy or lymphoma or lung cancer or breast cancer or myocardial infarction or gi perforation$ or gastrointestinal perforation$ or neutropenia or anaemia or anemia or dili or (drug induced adj2 liver injur$)).ti,ab. (3365623)

20 7 or 8 or 9 or 10 or 11 or 12 or 13 or 14 or 15 or 16 or 17 (201918)

21 5 and 19 and 20 (1856)

22 (cost$ or economic$ or budget).ti. (291671)

23 21 not 22 (1812)

24 23 not (phase adj ("1" or i or "2" or ii)).ti. (1801)

25 limit 24 to english language (1559)

26 remove duplicates from 25 (876)

27 26 not case report.ti. (859)

28 27 not (editorial or comment or letter or case report$ or interview or note).pt,lt. (732)

29 28 not (conference or meeting).pt,lt. (508)

30 29 and ((study or trial).ti. or (clinical or trial$).ab. or ((clin$ or patient$ or subject$) adj2 (stud$ or trial$)).mp.) (348) additional dups removed manually in EndNote = 330 total

Manual addition of 13 articles; total=343 records

# INDIVIDUAL TRIAL CHARACTERISTICS FOR ARTICLES USED IN THE meta-ANALYSIS

Table 1. Trials of biologic disease-modifying antirheumatic drugs and tofacitinib in patients with moderate to severely active rheumatoid arthritis who were included in the serious infections analyses

| **Author (study name), year** | **Study design** | **Study duration** | **Patients (N)** | **Age**  **(years)** | **Proportion of females (%)** | **Baseline CRP (mg/L)** | **Level of evidence** | **Prior treatment** | **Jadad Scale** |
| --- | --- | --- | --- | --- | --- | --- | --- | --- | --- |
| **Abatacept studies** |  |  |  |  |  |  |  |  |  |
| Bathon (AGREE), 2011 [1] | Phase 3b RCT | 2 years | 509 | 49.2-49.9 | 76-78 | 31.0-36.0 | 1b | MTX-naive | 4 |
| Kremer (AIM), 2006 [2] | Phase 3 RCT | 1 year | 652 | 50.4-51.5 | 78-82 | 28.0-33.0 | 1b | MTX-IR | 5 |
| Weinblatt (AMPLE), 2013 [3] | Phase 3b RCT | 1 year | 646 | 51.0-51.4 | 81-82 | 15.0-16.0 | 1b | MTX-IR | 2 |
| Genovese (ACQUIRE), 2011 [4] | Phase 3b RCT | 6 months | 1457 | 49.9-50.1 | 80-84 | 26.0-27.0 | 1b | MTX-IR | 4 |
| Genovese (ATTAIN), 2005 [5] | Phase 3 RCT | 6 months | 393 | 52.7-53.4 | 77-80 | 40.0-46.0 | 1b | TNFi-IR | 5 |
| Schiff (ATTEST), 2008 [6] | Phase 3 RCT | 1 year | 748 | 49.0-49.4 | 82-87 | 27.0-33.0 | 1b | MTX-IR | 4 |
| Kremer, 2005 [7] | Phase 2b RCT | 1 year | 339 | 54.4-55.8 | 63-75 | 29.0-32.0 | 2b | MTX-IR | 5 |
| Genovese (ATTAIN), 2008 [8] | OL LTE | 2 years | 391 | 52.7-53.1 | 77-80 | 33.0-46.0 | 2b | TNFi-IR | n/a |
| Kremer (AIM), 2008 [9] | OL LTE | 2 years | 547 | 49.5-51.4 | 77-85 | 25.0-32.0 | 2b | MTX-IR | n/a |
| Westhovens, 2009 [10] | OL LTE | 5 years | 219 | NA | NA | NA | 2b | MTX-IR | n/a |
| **Adalimumab studies** |  |  |  |  |  |  |  |  |  |
| Gabay (ADACTA), 2013 [11] | Phase 4 RCT | 24 weeks | 326 | 53.3-54.4 | 79-82 | 25.0-26.0 | 2b | MTX-IR | 5 |
| Weinblatt (AMPLE), 2013 [3] | Phase 3b RCT | 1 year | 646 | 51.0-51.4 | 81-82 | 15.0-16.0 | 1b | MTX-IR | 2 |
| Weinblatt (ARMADA), 2003 [12] | Phase 2/3 RCT | 24 weeks | 271 | 53.5-57.2 | 75-82 | 21.0-31.0 | 1b | MTX-IR | 3 |
| Weinblatt (ARMADA 4 yr), 2006 [13] | OL LTE | 4 years | 262 | 55.0 | 76 | 26.0 | 2b | MTX-IR | n/a |
| Miyasaka (CHANGE)^†^, 2008 [14] | Phase 2/3 RCT | 24 weeks | 352 | 53.4-56.9 | 77-83 | 49.7-65.6 | 1b | csDMARD-IR | 4 |
| van de Putte (DE011)^†^, 2004 [15] | Phase 3 RCT | 26 weeks | 544 | 51.8-54.4 | 72-80 | 47.2-57.0 | 2b | csDMARD-IR | 5 |
| Keystone (DE019), 2004 [16] | Phase 3 RCT | 1 year | 619 | 56.1-57.3 | 73-76 | 14.0-18.0 | 1b | MTX-IR | 4 |
| Detert (HIT HARD), 2013 [17] | Phase 4 RCT | 48 weeks | 179 | 47.2-52.5 | 67-70 | 12.0-17.0^*^ | 2b | DMARD-naive | 4 |
| Keystone (OL of DE019), 2011 [18] | OL LTE | 5 years | 553 | 55.7 | 75 | 17.0 | 2b | MTX-IR | n/a |
| Kavanaugh (OPTIMA), 2013 [19] | Phase 4 RCT | 26 weeks | 1032 | 50.4-50.7 | 74 | 27.0-30.0 | 1b | MTX-naive | 5 |
| Breedveld (PREMIER), 2006 [20] | Phase 3 RCT | 2 years | 799 | 51.9-52.1 | 72-77 | 39.0-41.0 | 1b | MTX-naive | 3 |
| van der Heijde (PREMIER 5 yr), 2010 [21] | OL LTE | 5 years | 497 | NA | NA | NA | 2b | MTX-naive | n/a |
| Burmester (ReAct), 2007 [22] | Prospective, OL | 12 weeks | 6610 | 54.0 | 81 | 26.0 | 2b | DMARD-IR | n/a |
| van Vollenhoven (ORAL Standard), 2012 [23] | Phase 3 RCT | 1 year | 717 | 51.9-55.5 | 75-85 | 11.6-20.3 | 1b | MTX-IR | 5 |
| Furst (STAR), 2003 [24] | Phase 2 RCT | 24 weeks | 636 | 55.0-55.8 | 79-80 | 15.0 | 1b | csDMARD-IR | 4 |
| van de Putte^†^, 2003 [25] | Phase 2 RCT | 12 weeks | 284 | 50.2-53.7 | 69-85 | 56.0-63.0 | 1b | csDMARD-IR | 4 |
| Rau, 2004 [26] | Phase 1 RCT | 42.2 days | 54 | 52.3-54.1 | 61-89 | 23.0-33.0 | 1b | MTX-IR | 4 |
| Kim, 2007 [27] | Phase 3 RCT | 24 weeks | 128 | 48.5-49.8 | 86-95 | 22.0-27.0 | 2b | csDMARD-IR | 4 |
| Bejarano, 2008 [28] | Phase 4 RCT | 56 weeks | 148 | 47.0 | 53-58 | 28.7-38.2 | 2b | MTX-naive | 5 |
| Chen, 2009 [29] | Phase 2 RCT | 12 weeks | 47 | 53.0 | 74-92 | 20.0-24.0 | 2b | MTX-IR | 3 |
| Fleischmann (A3921035)^†^, 2012 [30] | Phase 2b RCT | 24 weeks | 386 | 52.0-55.0 | 85-88 | 16.2-24.5 | 1b | csDMARD-IR | 4 |
| **Certolizumab studies** |  |  |  |  |  |  |  |  |  |
| Fleischmann (FAST4WARD)^†^, 2009 [31] | Phase 3 RCT | 24 weeks | 220 | 52.7-54.9 | 78-89 | 11.3-11.6 | 2b | csDMARD-IR | 5 |
| Keystone (RAPID 1), 2008 [32] | Phase 3 RCT | 1 year | 982 | 51.4-52.4 | 82-84 | 14.0-16.0^*^ | 1b | MTX-IR | 3 |
| Smolen (RAPID 2), 2009 [33] | Phase 3 RCT | 24 weeks | 619 | 51.5-52.2 | 78-84 | 13.5-14.2 | 2b | MTX-IR | 4 |
| Weinblatt (REALISTIC), 2012 [34] | Phase 3 RCT | 12 weeks | 1063 | 53.9-55.4 | 78-80 | 9.00-10.0^*^ | 1b | Mixed | 5 |
| Choy, 2012 [35] | Phase 3 RCT | 24 weeks | 247 | 53.0-55.6 | 66-72 | 11.9-13.1 | 1b | MTX-IR | 5 |
| **Etanercept studies** |  |  |  |  |  |  |  |  |  |
| Emery (COMET), 2008 [36] | Phase 4 RCT | 2 years | 542 | 50.5-52.3 | 73-74 | 36.5-37.0 | 2b | DMARD-naive | 5 |
| Emery (COMET), 2010 [37] | Phase 4 RCT | 2 years | 411 | 52.2-55.6 | 59-82 | 6.00-10.5 | 2b | DMARD-naive | 5 |
| Genovese (ERA), 2002 [38] | OL LTE | 2 years | 632 | 49.0-51.0 | 74-75 | 33.0-44.0 | 2b | MTX-naive | n/a |
| Genovese (ERA 5 yr), 2005 [39] | OL LTE | 5 years | 632 | 48.3-49.9 | 73-77 | 20.1-25.3 | 2b | MTX-naive | n/a |
| Kameda (JESMR), 2011 [40] | Phase 4, prospective OL study | 1 year | 151 | 56.6-58.1 | 80-87 | 25.0-30.0 | 2b | MTX-IR | 3 |
| Klareskog (TEMPO), 2004 [41] | Phase 3 RCT | 1 year | 686 | 52.5-53.2 | 74-79 | 25.5-32.4 | 2b | csDMARD-IR | 5 |
| van der Heijde (TEMPO 2 yr), 2006 [42] | RCT, LTE | 2 years | 686 | 52.5-53.2 | 74-79 | 25.5-32.4 | 2b | csDMARD-IR | 4 |
| van der Heijde (TEMPO 3 yr), 2007 [43] | RCT, LTE | 3 years | 686 | 52.5-53.2 | 74-79 | 25.5-32.4 | 1b | csDMARD-IR | 3 |
| Study 20000125, 1998 [44] | Phase 2 OL single arm study | 24 weeks | 58 | 48.9 | 85 | 21.9 | 2b | TNFi-IR | n/a |
| Lan, 2004 [45] | Phase 2 RCT | 12 weeks | 58 | 47.6-50.8 | 83-90 | 16.5-18.3 | 1b | MTX-IR | 3 |
| Genovese, 2004 [46] | RCT | 6 months | 244 | 53.8-55.7 | 72-83 | 20.0-24.0 | 2b | MTX-IR | 4 |
| Bliddal, 2006 [47] | RCT | 1 day | 39 | 53 | NA | NA | 1b | csDMARD-IR | 5 |
| Combe, 2006 [48] | RCT | 24 weeks | 260 | 50.6-53.3 | 79-82 | 11.6-14.3^*^ | 1b | csDMARD-IR | 3 |
| Klareskog, 2006 [49] | OL LTE | 5 years | 549 | 53.0 | 79 | 43.4 | 2b | csDMARD-IR | n/a |
| Weinblatt, 2007 [50] | Phase 2b RCT | 2 years | 121 | 49.8-54.3 | 72-78 | 20.0-24.0 | 2b | bDMARD-IR | 5 |
| Dore, 2007 [51] | Phase 3 OL single arm study | 24 weeks | 223 | 53.4 | 81 | NA | 2b | MTX-IR | n/a |
| Weinblatt, 2008 [52] | Phase 4 RCT | 24 weeks | 201 | 51.1-53.0 | 70-84 | 8.90-9.40 | 1b | TNFi-IR | 3 |
| Klareskog, 2011 [53] | OL LTE | 5 years | 549 | NA | NA | NA | 2b | csDMARD-IR | n/a |
| **Golimumab studies** |  |  |  |  |  |  |  |  |  |
| Smolen (GO-AFTER), 2009 [54] | Phase 3 RCT | 24 weeks | 461 | 54.0-55.0^*^ | 74-85 | 8.00-10.0^*^ | 1b | TNFi-IR | 5 |
| Emery (GO-BEFORE), 2009 [55] | Phase 3 RCT | 1 year | 637 | 48.2-50.9 | 79-85 | 24.0-26.0 | 1b | MTX-naive | 5 |
| Keystone (GO-FORWARD), 2009 [56] | Phase 3 RCT | 1 year | 444 | 50.0-52.0^*^ | 79-82 | 8.00-10.0^*^ | 1b | MTX-IR | 5 |
| Keystone (GO-FORWARD), 2010 [57] | Phase 3 RCT LTE | 1 year | 444 | 50.0-52.0^*^ | 79-82 | 8.00-10.0^*^ | 1b | MTX-IR | 5 |
| Weinblatt (GO-FURTHER), 2013 [58] | Phase 3 RCT | 100 weeks | 592 | 51.4-51.9 | 80-83 | 22.0-28.0 | 2b | MTX-IR | 5 |
| Kay, 2008 [59] | Phase 2 RCT | 1 year | 172 | 48.0-57.5^*^ | 68-86 | 14.0-21.0^*^ | 2b | MTX-IR | 4 |
| **Infliximab studies** |  |  |  |  |  |  |  |  |  |
| Schiff (ATTEST), 2008 [6] | Phase 3 RCT | 1 year | 748 | 49.0-49.4 | 82-87 | 27.0-33.0 | 1b | MTX-IR | 4 |
| Maini (ATTRACT), 1999 [60] | Phase 3 RCT | 30 weeks | 428 | 51.0-56.0^*^ | 73-81 | 20.0-31.0^*^ | 2b | MTX-IR | 4 |
| Lipsky (ATTRACT 1 yr), 2000 [61] | Phase 3 RCT | 54 weeks | 428 | 51.0-54.0 | 73-81 | 33.0-42.0 | 2b | MTX-IR | 4 |
| Maini (ATTRACT 2 yr), 2004 [62] | OL LTE | 102 weeks | 428 | 51.0-56.0 | 73-81 | 20.0-31.0^*^ | 2b | MTX-IR | n/a |
| Fleischmann (iRAMT), 2005 [63] | OL, single arm | 46 weeks | 210 | 53.2 | 74 | 18.7 | 2b | MTX-IR | n/a |
| Westhovens (START), 2006 [64] | Phase 3 RCT | 1 year | 1084 | 52.0-53.0^*^ | 78-83 | 12.0-16.0^*^ | 1b | MTX-IR | 5 |
| St Clair (ASPIRE), 2004 [65] | Phase 3 RCT | 54 weeks | 1049 | 50.0-51.0 | 68-75 | 26.0-30.0 | 1b | MTX-naive | 5 |
| Durez, 2004 [66] | RCT | 6 weeks | 27 | 48.0-56.0 | 73-100 | 13.0-19.0^*‡^ | 2b | csDMARD-IR | 1 |
| Durez, 2007 [67] | Phase 4 RCT | 1 year | 44 | 50.0-53.8 | 60-71 | 25.0-48.0 | 1b | MTX-naive | 2 |
| Pavelka, 2009 [68] | Phase 4 RCT | 1 year | 141 | 45.6-48.1 | 67-69 | 37.0 | 2b | csDMARD-IR | 5 |
| Takeuchi (RISING), 2009 [69] | Phase 3 RCT | 1 year | 334 | 48.8-50.4 | 79-86 | 30 | 1b | MTX-IR | 4 |
| **Rituximab studies** |  |  |  |  |  |  |  |  |  |
| Emery (DANCER), 2006 [70] | Phase 2b RCT | 24 weeks | 465 | 51.1-51.4 | 80-83 | 30.0-33.0 | 1b | MTX-IR | 4 |
| Tak (IMAGE), 2011 [71] | Phase 3 RCT | 1 year | 755 | 47.9-48.1 | 77-85 | 30.0-34.0 | 1b | MTX-naive | 5 |
| Tak (IMAGE 2 yr), 2012 [72] | Phase 3 RCT LTE | 2 years | 755 | 47.9-48.1 | 77-85 | 30.0-34.0 | 1b | MTX-naive | 4 |
| Rubbert-Roth (MIRROR), 2010 [73] | Phase 3 RCT | 48 weeks | 378 | 51.3-53.6 | 76-83 | 21.0-26.0 | 1b | DMARD-IR | 5 |
| Cohen (REFLEX), 2006 [74] | Phase 3 RCT | 24 weeks | 520 | 52.2-52.8 | 81 | 37.0-38.0 | 2b | TNFi-IR | 4 |
| Emery (SERENE), 2010 [75] | Phase 3 RCT | 48 weeks | 511 | 51.3-52.2 | 80-86 | NA | 1b | MTX-IR | 4 |
| Mease (SUNRISE), 2010 [76] | Phase 3 RCT | 48 weeks | 475 | 54.0 | 79-81 | 19.0-22.0 | 1b | TNFi-IR | 4 |
| Edwards, 2004 [77] | Phase 2 RCT | 24 weeks | 161 | 53.0-54.0 | 73-83 | 26.0-40.0 | 1b | MTX-IR | 4 |
| **Tocilizumab studies** |  |  |  |  |  |  |  |  |  |
| Gabay (ADACTA), 2013 [11] | Phase 4 RCT | 24 weeks | 326 | 53.3-54.4 | 79-82 | 25.0-26.0 | 2b | MTX-IR | 5 |
| Jones (AMBITION), 2010 [78] | Phase 3 RCT | 24 weeks | 673 | 50.0-50.7 | 79-83 | 30.0-31.0 | 1b | MTX-naive | 3 |
| Maini (CHARISMA), 2006 [79] | Phase 2 RCT | 20 weeks | 359 | 49.2-52.2 | 73-87 | 19.0-32.0 | 1b | MTX-IR | 5 |
| Kremer (LITHE), 2011 [80] | Phase 3, RCT | 1 year | 1190 | 51.3-53.4 | 82-84 | 21.0-23.0 | 1b | MTX-IR | 3 |
| Fleischmann LITHE (2 yr), 2013 [81] | Phase 3 RCT LTE | 2 years | 1190 | 51.3-53.4 | 82-84 | 21.0-23.0 | 1b | MTX-IR | 4 |
| Smolen (OPTION), 2008 [82] | Phase 3 RCT | 24 weeks | 623 | 50.6-51.4 | 78-85 | 24.0-28.0 | 1b | MTX-IR | 5 |
| Emery (RADIATE), 2008 [83] | Phase 3 RCT | 24 weeks | 499 | 50.9-53.9 | 79-84 | 28.0-37.1 | 1b | TNFi-IR | 4 |
| Yazici (ROSE), 2012 [84] | Phase 3b RCT | 24 weeks | 619 | 55.2-55.8 | 80-84 | 18.0-18.3 | 1b | DMARD-IR | 4 |
| Nishimoto (SAMURAI), 2007 [85] | Phase 3 RCT | 1 year | 306 | 52.9-53.1 | 80-82 | 47.0-49.0 | 1b | csDMARD-IR | 3 |
| Nishimoto (SATORI), 2009 [86] | Phase 3 RCT | 24 weeks | 125 | 50.8-52.6 | 75-90 | 30.0-32.0 | 2b | MTX-IR | 5 |
| Nishimoto (STREAM), 2009 [87] | OL LTE | 5 years | 143 | 54.3 | 76 | 47.0 | 2b | csDMARD-IR | n/a |
| Burmester (TAMARA), 2011 [88] | Phase 3b OL study | 24 weeks | 286 | NA | 76 | 20.7-26.7 | 2b | DMARD-IR | n/a |
| Genovese (TOWARD), 2008 [89] | Phase 3 RCT | 24 weeks | 1220 | 53.0-54.0 | 81-84 | 26.0 | 1b | csDMARD-IR | 4 |
| **Tofacitinib studies** |  |  |  |  |  |  |  |  |  |
| Lee (ORAL Start), 2014 [90] | Phase 3 RCT | 2 years | 956 | 48.8-50.3 | 77-82 | 20.3-25.9 | 1b | MTX-naive | 4 |
| McInnes (A3921109), 2014 [91] | Phase 2  RCT | 12 weeks | 111 | 52.0 | 90 | 22.2-33.2 | 1b | DMARD-IR | n/a |
| Kremer (A3921025)^§^, 2012 [92] | Phase 2b RCT | 24 weeks | 509 | 51.0-56.0 | 74-88 | 14.4-18.9 | 1b | MTX-IR | 3 |
| van der Heijde (ORAL Scan), 2013 [93] | Phase 3 RCT | 2 years | 800 | 52.0-53.7 | 84-91 | 12.2-17.0 | 1b | MTX-IR | 5 |
| Fleischmann (ORAL Solo)^†^, 2012 [94] | Phase 3 RCT | 6 months | 611 | 49.7-52.4 | 85-88 | 17.8-22.9 | 1b | DMARD-IR | 5 |
| van Vollenhoven (ORAL Standard), 2012 [23] | Phase 3 RCT | 1 year | 717 | 51.9-55.5 | 75-85 | 11.6-20.3 | 1b | MTX-IR | 5 |
| Burmester (ORAL Step), 2013 [95] | Phase 3 RCT | 6 months | 399 | 54.4-55.4 | 80-87 | 15.7-19.3 | 1b | TNFi-IR | 5 |
| Kremer (ORAL Sync), 2013 [96] | Phase 3 RCT | 1 year | 795 | 51.9-53.3 | 75-84 | 16.5-17.7 | 1b | DMARD-IR | 5 |
| Tanaka (A3921039)^§^, 2011 [97] | Phase 2 RCT | 12 weeks | 140 | 50.0-53.3 | 75-96 | 16.6-27.6 | 1b | MTX-IR | 4 |
| Fleischmann (A3921035)^†§^, 2012 [30] | Phase 2b RCT | 24 weeks | 386 | 52.0-55.0 | 85-88 | 16.2-24.5 | 1b | DMARD-IR | 4 |
| Tanaka (A3921040)^†§^, 2014 [98] | Phase 2b RCT | 12 weeks | 317 | 52.8-54.7 | 79-89 | 16.6-27.6 | 1b | DMARD-IR | 5 |
| Active comparator studies may be listed under both therapies  ^*^Median values reported; n/a=not applicable ^†^Monotherapy studies in MTX-IR ^‡^Serum CRP values presented in grams per liter  ^§^Data shown are from all treatment arms; only data for tofacitinib 5 and 10 mg BID, placebo, MTX, adalimumab monotherapy, and adalimumab + MTX were included in the meta-analysis  1b, randomized, blinded trial with incidence rates for serious infection available, or sufficient information (number of patients, number or % of patients with event and <20% dropout rate) to calculate incidence rates  2b, other trials (eg open-label, long-term extension studies) that comply with the inclusion/exclusion criteria  BID, twice daily; CRP, C-reactive protein; DMARD, disease-modifying antirheumatic drug; DMARD-IR, disease-modifying antirheumatic drug inadequate responder (includes bDMDARDs and csDMARDs); bDMARD-IR, biologic DMARD inadequate responder; csDMARD-IR, conventional synthetic DMARD inadequate responder; LTE, long-term extension; MTX, methotrexate; MTX-IR, methotrexate inadequate responder; NA, not available; OL, open-label; RCT, randomized controlled trial; TNFi-IR, tumor necrosis factor inhibitor inadequate responder | | | | | | | | | |

# Summary of risk ratio and risk difference results across biologic dmards and tofacitinib in methotrexate naIve trials

| Table 2. Risk ratios and risk differences for rates of serious infection events associated with approved biologic DMARDs and tofacitinib in randomized clinical trials conducted in methotrexate-naive patients | | | | | |
| --- | --- | --- | --- | --- | --- |
| Agent | Studies included | Sample size (agent, MTX) | RR  (95% CI) | RD %  (95% CI) | References |
| Abatacept | 1 | 509  (256, 253) | 0.99  (0.29, 3.37) | -0.02 (-2.43, 2.39) | AGREE [1] |
| Rituximab | 1 | 504  (252, 252) | 0.46  (0.18, 1.20) | -2.78  (-6.09, 0.54) | IMAGE [71] |
| Tocilizumab | 1 | 572 (288, 284) | 1.97  (0.36, 10.68) | 0.68  (-0.98, 2.35) | AMBITION [78] |
| Adalimumab | 4 | 1877  (945, 932) | 1.43  (0.80, 2.56) | 1.07  (-0.29, 2.42) | PREMIER [20]  Bejarano 2008 [28] HIT HARD [17] OPTIMA [19] |
| Etanercept | 3 | 1425  (712, 713) | 0.82  (0.47, 1.43) | -0.79  (-2.63, 1.04) | ERA [38]  TEMPO [42]  COMET [36] |
| Infliximab | 2 | 700  (388, 312) | 2.80  (1.18, 6.61) | 3.70  (0.91, 6.49) | ASPIRE [65] Durez 2007 [66] |
| Golimumab | 1 | 318  (158, 160) | 0.68  (0.11, 3.99) | -0.61  (-3.34, 2.12) | GO-BEFORE [55] |
| TNFi | 10 | 4320  (2203, 2117) | 1.24  (0.87, 1.77) | 0.65  (-0.33, 1.63) | ERA [38]  TEMPO [42]  ASPIRE [65]  PREMIER [20]  Durez 2007 [66]  Bejarano 2008 [28]  COMET [36] HIT HARD [17]  OPTIMA [19] GO-BEFORE [55] |
| Tofacitinib 5 mg BID | 1 | 559  (373, 186) | 1.1  (0.39, 3.11) | 0.26  (-2.63, 3.15) | ORAL Start [90] |
| Tofacitinib 10 mg BID | 1 | 583  (397, 186) | 0.75  (0.25, 2.26) | -0.67%  (-3.38, 2.03) | ORAL Start [90] |
| BID, twice daily; CI, confidence interval; DMARD, disease-modifying antirheumatic drug; MTX, methotrexate;  RD, risk difference; RR, risk ratio; TNFi, tumor necrosis factor inhibitors | | | | | |

# summary of age, gender and study duration by drug

| Table 3. Summary of age, gender and study duration by drug | | | | |
| --- | --- | --- | --- | --- |
| Drug | Number of studies included | Mean patient age at baseline,  years (range) | Median study duration,  weeks (range) | Mean proportion of female patients,  % (range) |
| TNF inhibitors | 57 | 52.0 (45.6, 57.5) | 52 (12, 260) | 78.1 (58.4, 95.4) |
| Abatacept | 11 | 50.4 (49.0, 53.4) | 99 (26, 260) | 79.5 (75.0, 84.4) |
| Rituximab | 8 | 50.6 (47.9, 54) | 48 (24, 100) | 81.4 (73.0, 85.0) |
| Tocilizumab | 13 | 52.6 (50.1, 55.2) | 24 (20, 260) | 81.4 (75.5, 90.2) |
| Tofacitinib | 10 | 52.9 (49.0, 56) | 52 (12, 100) | 83.3 (55.0, 96.2) |
| TNF inhibitors included adalimumab, certolizumab, etanercept, golimumab and infliximab  TNF, tumor necrosis factor | | | | |

# Sensitivity Analyses TABLES

## Incidence Rate

| Table 4. Sensitivity analysis for contextualization: IR (95% CI) [N] | | | | | |
| --- | --- | --- | --- | --- | --- |
| Drug | Reference case | Case 1 | Case 2 | Case 3 | Case 4 |
| Abatacept | 3.04 (2.49, 3.72) [11] | 3.05 (2.37, 3.93) [12] | 2.39 (1.79, 3.18) [7] | 2.43 (1.80,3.27)  [7] | 2.75 (1.94, 3.90) [6] |
| Rituximab | 3.72 (2.99, 4.62) [8] | 3.01 (2.52, 3.60) [8] | 3.25 (2.57, 4.10) [7] | 3.32 (2.57, 4.30) [7] | 3.29 (2.37, 4.58) [7] |
| Tocilizumab | 5.45 (4.26, 6.96) [13] | 5.18 (4.16, 6.45) [13] | 5.25 (4.09, 6.75) [11] | 5.25 (4.09, 6.75) 11] | 5.64 (4.25, 7.49) [11] |
| Infliximab | 6.11 (5.24, 7.12) [11] | 5.88 (5.07, 6.83) [12] | 6.21 (5.34, 7.22) [11] | 6.02 (5.11, 7.10) [10] | 5.58 (4.47, 6.97) [10] |
| Etanercept | 4.06 (3.26, 5.08) [17] | 3.75 (2.95, 4.76) [18] | 3.76 (2.83, 5.00) [9] | 3.76 (2.83, 5.00) [9] | 3.65 (2.73, 4.87) [8] |
| Certolizumab pegol | 7.59 (5.80, 9.94) [5] | 7.44 (5.69, 9.73) [5] | 6.06 (4.69, 7.83) [4] | 6.06 (4.69, 7.83) [4] | 5.7 (3.82, 8.49)  [2] |
| Golimumab | 5.31 (4.09, 6.89) [6] | 4.81 (3.63, 6.37) [6] | 4.81 (3.63, 6.37) [6] | 4.39 (2.93, 6.56) [4] | 3.34 (1.81, 6.19) [4] |
| Adalimumab | 5.04 (3.80, 6.69) [18] | 4.75 (3.65, 6.19) [19] | 4.75 (3.42, 6.61) [14] | 4.75 (3.42, 6.61) [14] | 4.35 (2.58, 7.35) [11] |
| CI, confidence interval; IR, incidence rate; LTE, long-term extension; N, number of studies; RCT, randomized controlled trial  Reference = RCT + LTE studies, continuity factor, r = 0  Case 1 = RCT + LTE studies, continuity factor, r = 0.05  Case 2 = RCT studies only, continuity factor, r = 0.05  Case 3 = RCT studies, single record per study, continuity factor, r = 0.05  Case 4 = RCT studies only, FDA approved doses only, continuity factor, r = 0.05 | | | | | |

| Table 5. Sensitivity analysis using R 2.12.2 for trials with zero events. RR for approved biologics vs placebo in DMARD-IR population | | | | |
| --- | --- | --- | --- | --- |
| Drug | LCI | Mean | UCI | Number of Studies |
| Adalimumab | 0.82 | 2.00 | 4.85 | 11 |
| Tofacitinib 5 mg BID | 0.56 | 1.67 | 4.92 | 9 |
| Tofacitinib 10 mg BID | 0.55 | 1.62 | 4.72 | 9 |
| Certolizumab pegol | 1.06 | 2.18 | 4.50 | 5 |
| Rituximab | 0.46 | 1.01 | 2.22 | 5 |
| Tocilizumab | 1.21 | 1.81 | 2.71 | 9 |
| Abatacept | 0.50 | 1.19 | 2.80 | 4 |
| Golimumab | 0.43 | 1.30 | 3.90 | 4 |
| Etanercept | 0.07 | 1.00 | 15.24 | 1 |
| Infliximab | 0.35 | 0.84 | 1.97 | 3 |
| BID, twice daily; DMARD-IR, disease-modifying antirheumatic drug inadequate responder; LCI, lower confidence interval; RR, risk ratio; UCI, upper confidence interval | | | | |

Figure 1. Abatacept Incidence Rate


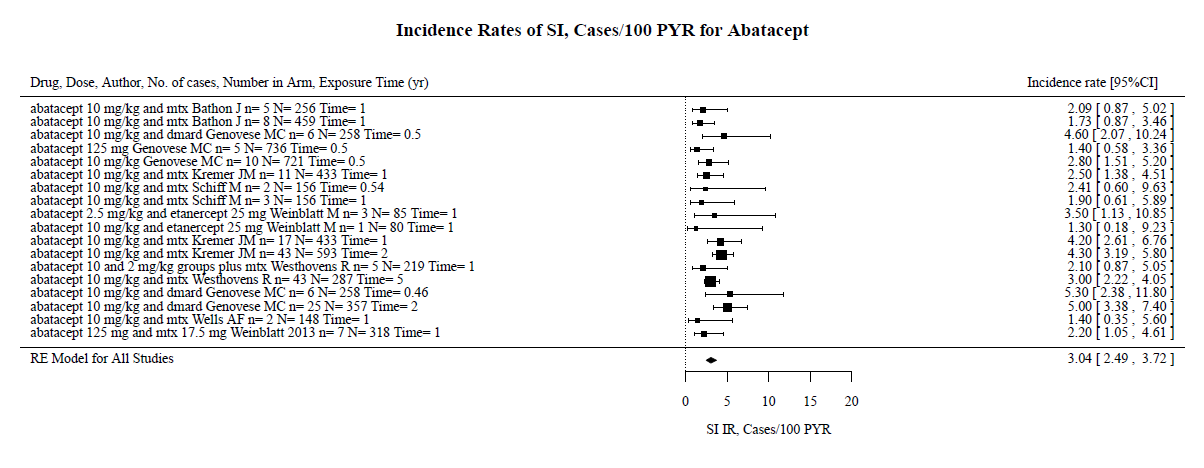


Figure 2. Rituximab Incidence Rate


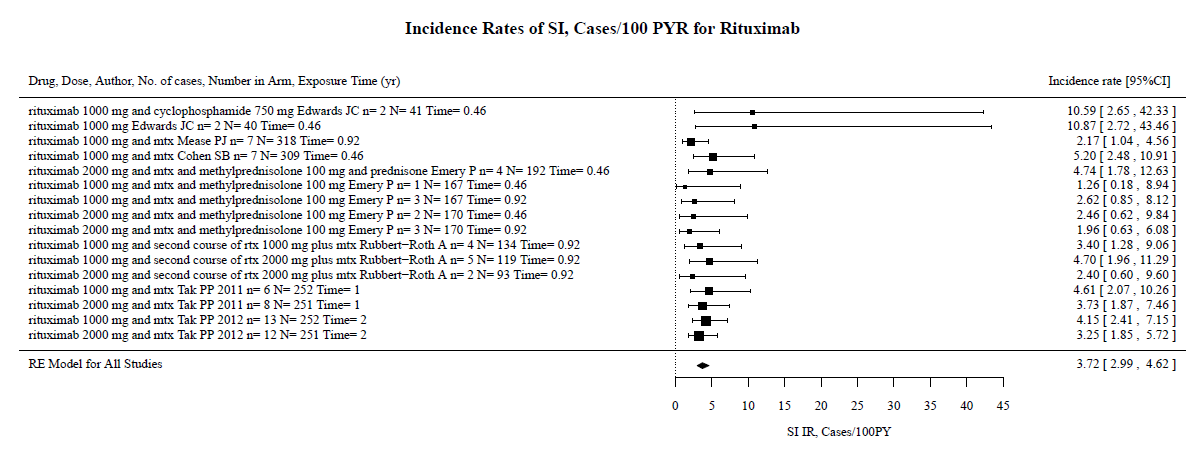


Figure 3. Tocilizumab Incidence Rate


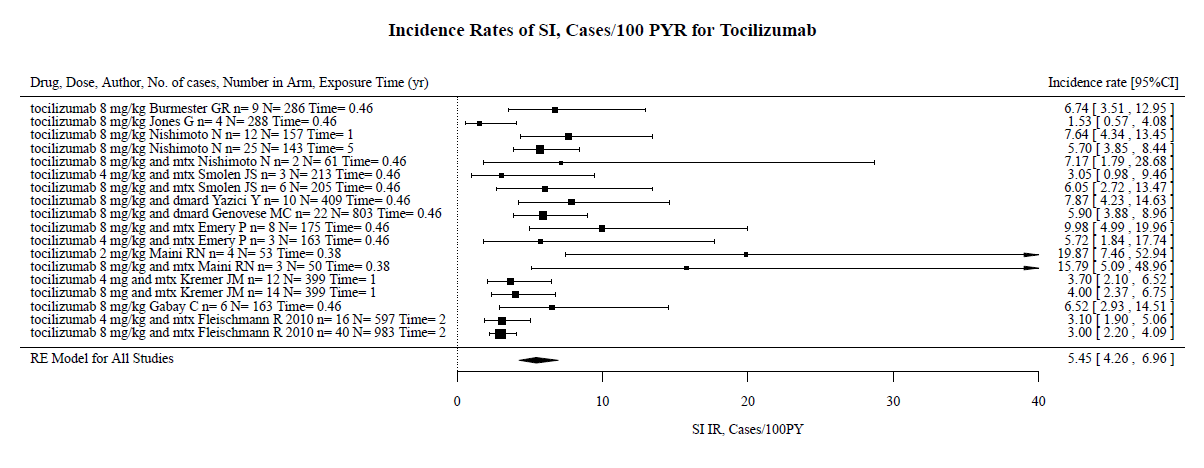


Figure 4. Infliximab Incidence Rate


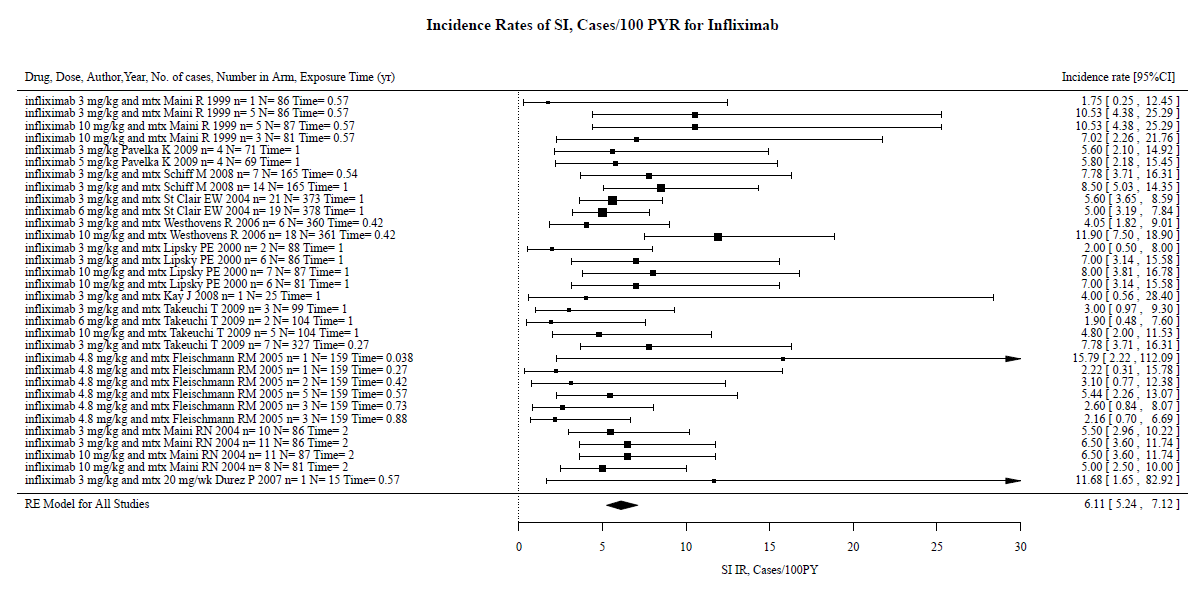


Figure 5. Etanercept Incidence Rate


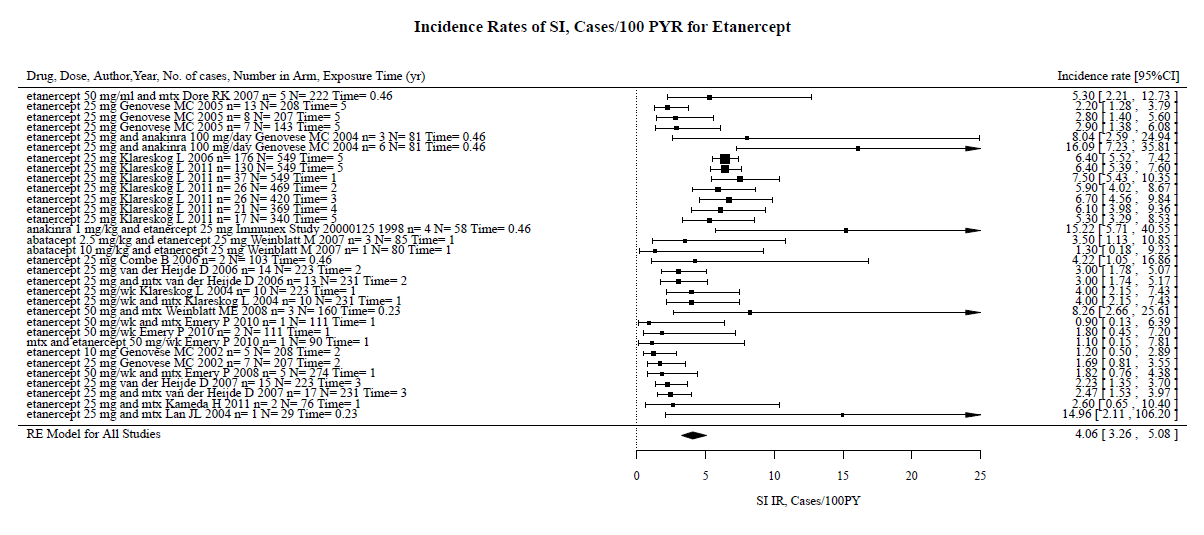


Figure 6. Certolizumab Pegol Incidence Rate


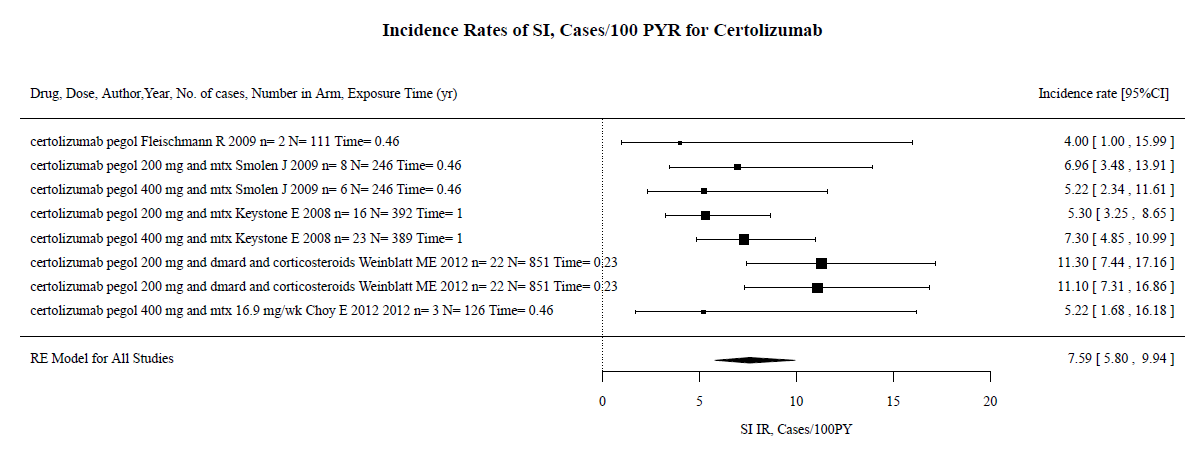


Figure 7. Golimumab Incidence Rate


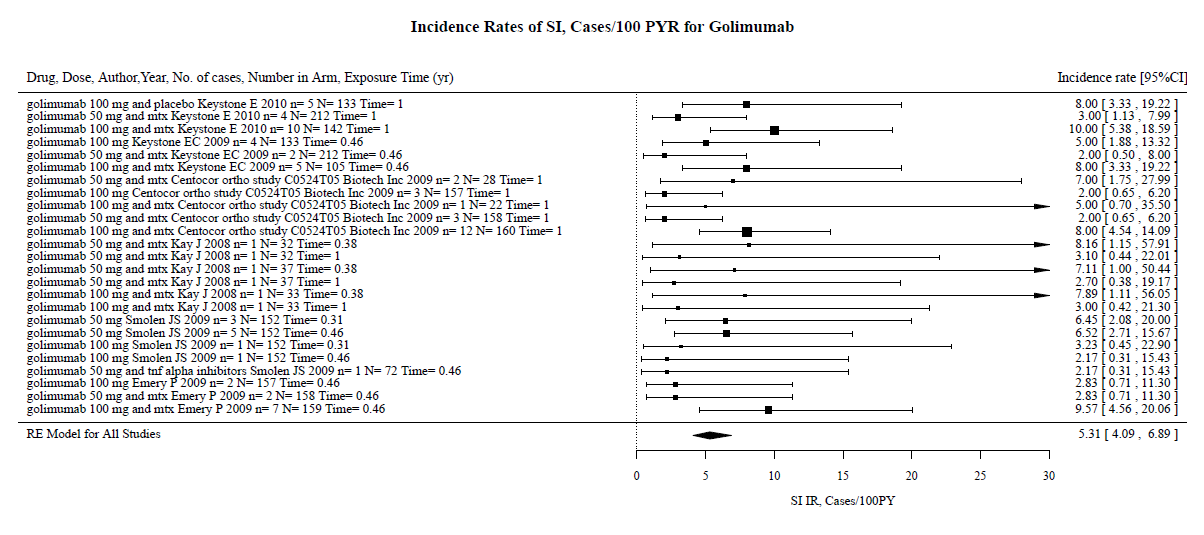


Figure 8. Adalimumab Incidence Rate


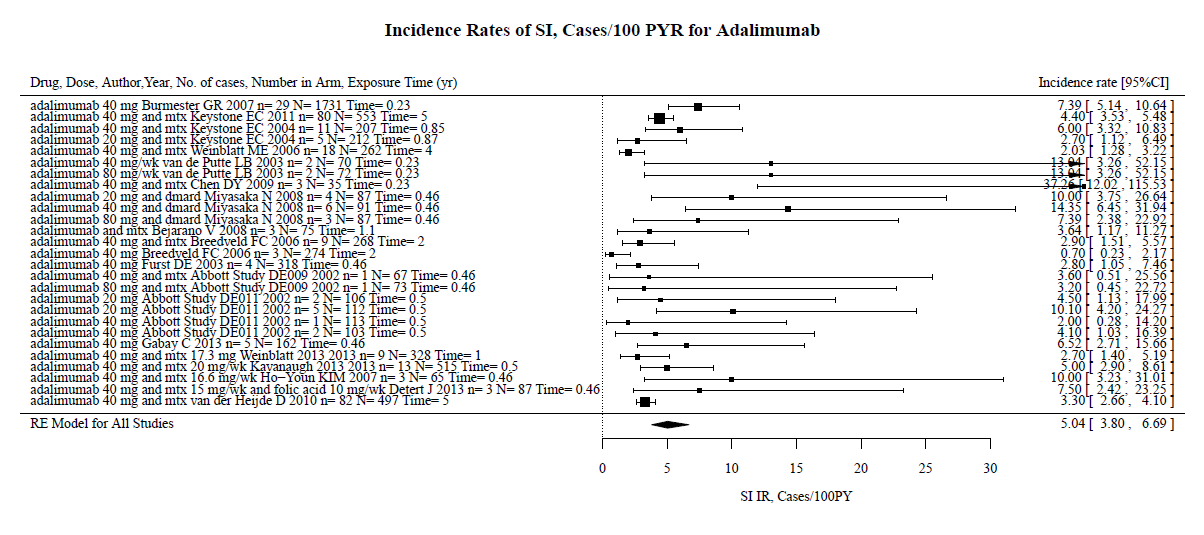


Figure 9. Incidence Rates of Adalimumab and Certolizumab from Monotherapy Trials in DMARD-IR Population


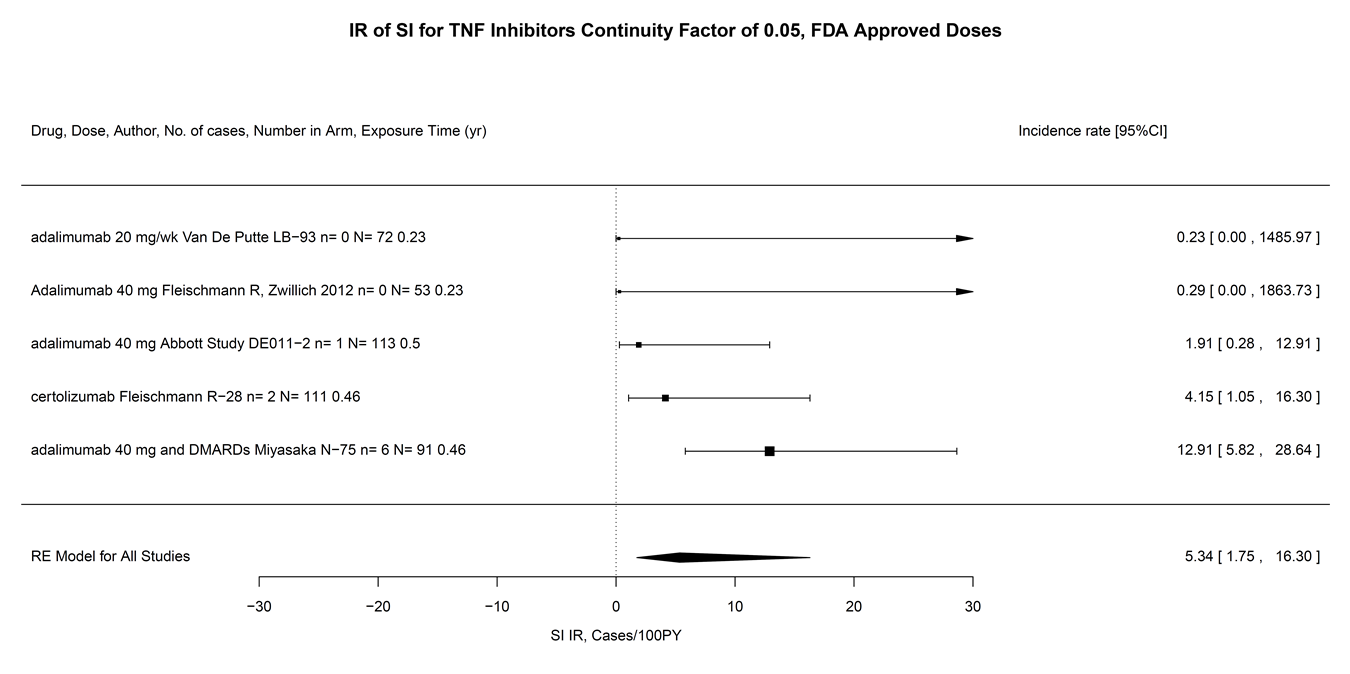


## Risk Ratio by Drug

### DMARD-IR Population

Figure 10. Abatacept Risk Ratio (DMARD-IR population)


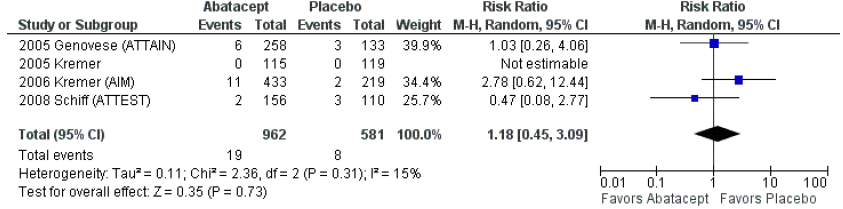


Figure 11. Rituximab Risk Ratio (DMARD-IR population)


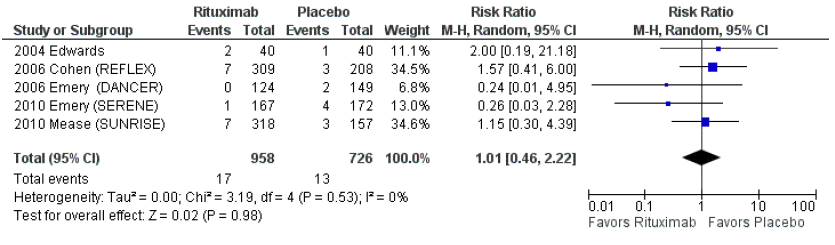


Figure 12. Tocilizumab Risk Ratio (DMARD-IR population)


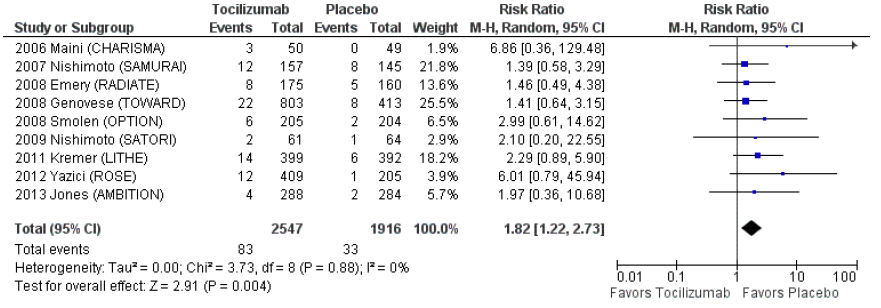


Figure 13. Infliximab Risk Ratio (DMARD-IR population)


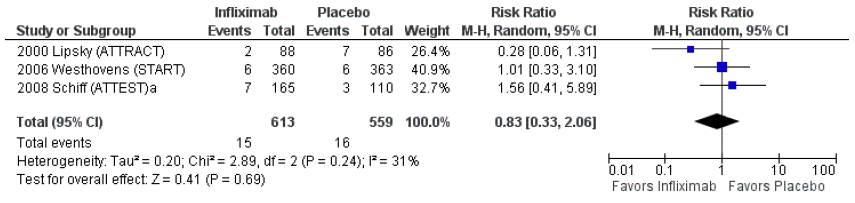


Figure 14. Golimumab Risk Ratio (DMARD-IR population)


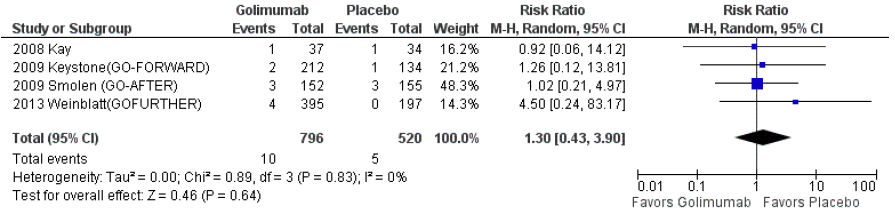


Figure 15. Certolizumab Risk Ratio (DMARD-IR population)


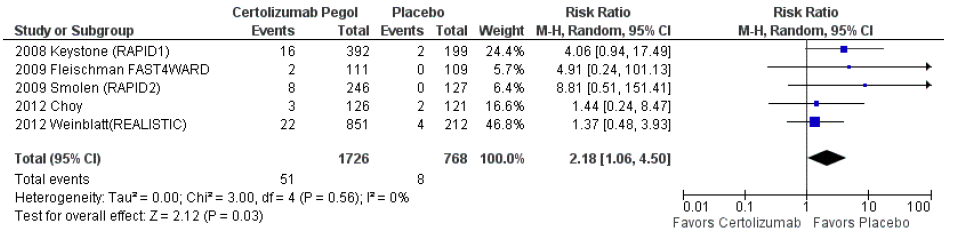


Figure 16. Adalimumab Risk Ratio (DMARD-IR population)


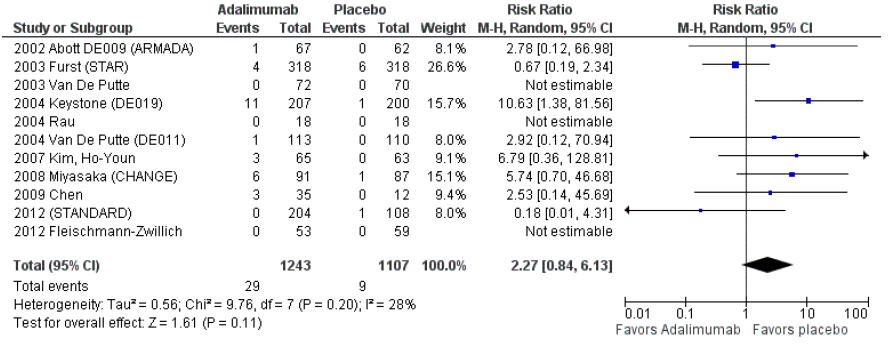


Figure 17. Etanercept Risk Ratio (DMARD-IR population)


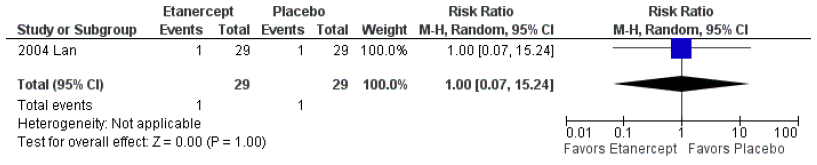


Figure 18. Tofacitinib 5 mg BID Risk Ratio (DMARD-IR population)


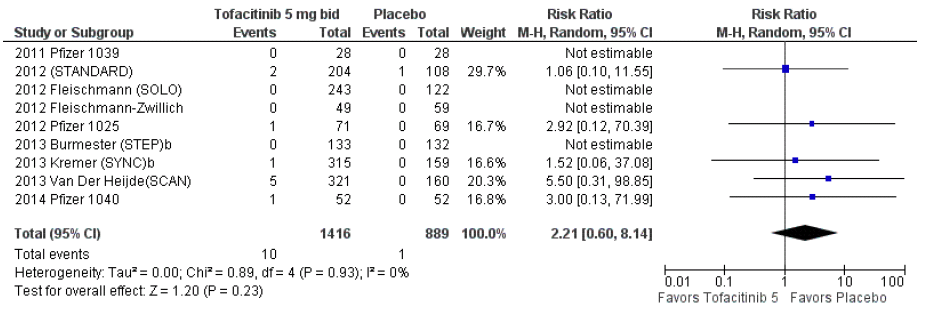


Figure 19. Tofacitinib 10 mg BID Risk Ratio (DMARD-IR population)


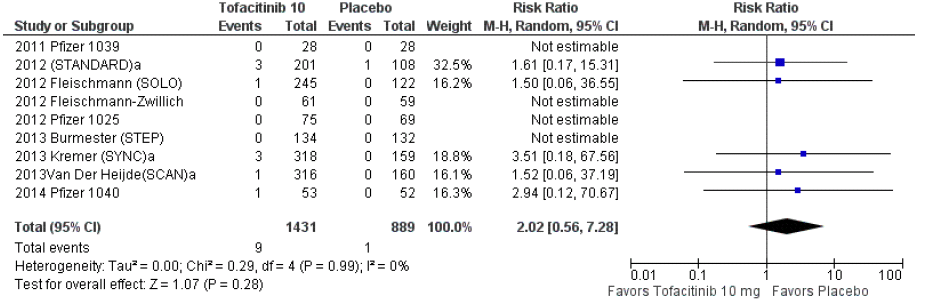


Figure 20. TNF Inhibitors Risk Ratio (DMARD-IR population)


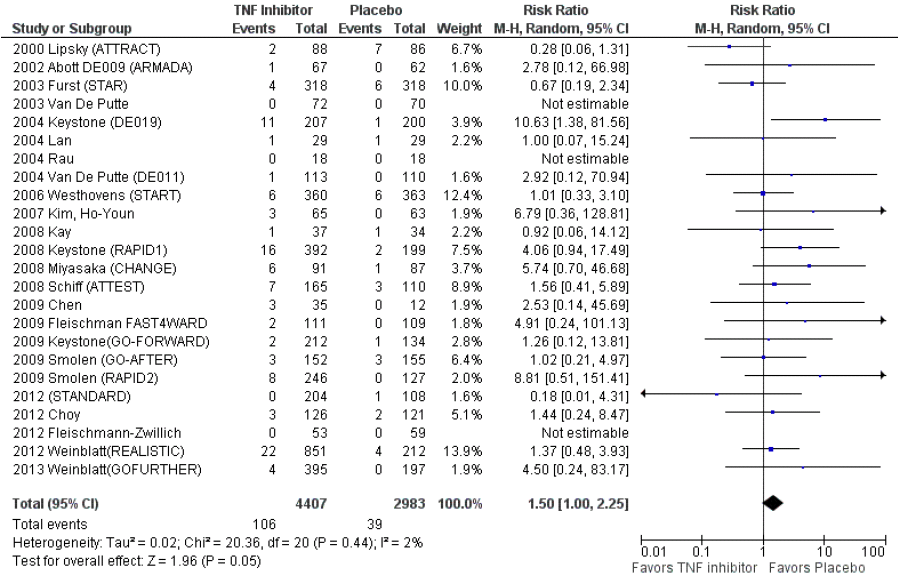


### MTX-naive Population

Figure 21. Abatacept Risk Ratio (MTX-naive population)


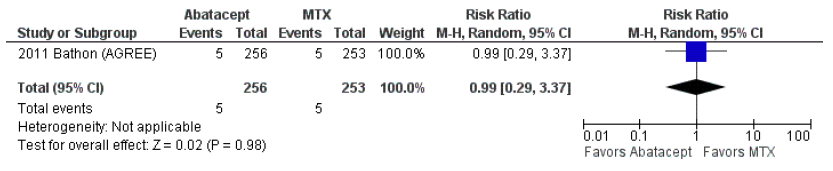


Figure 22. Rituximab Risk Ratio (MTX-naive population)


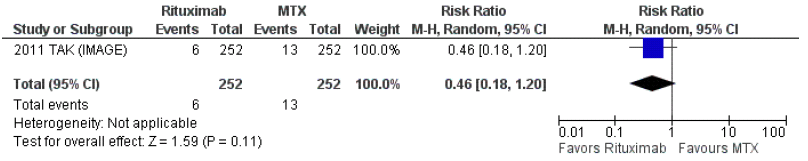


Figure 23. Infliximab Risk Ratio (MTX-naive population)


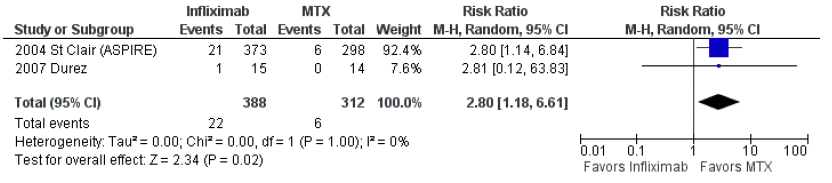


Figure 24. Golimumab Risk Ratio (MTX-naive population)


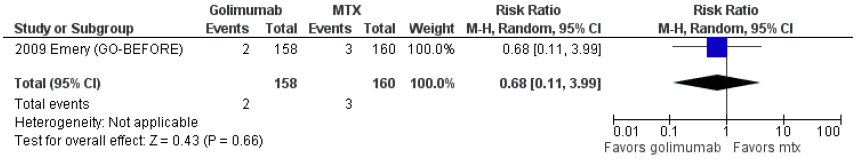


Figure 25. Adalimumab Risk Ratio (MTX-naive population)


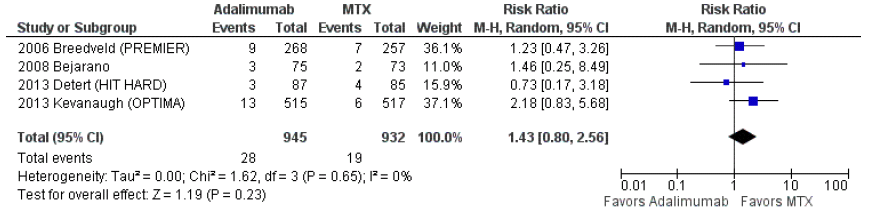


Figure 26. Etanercept Risk Ratio (MTX-naive population)


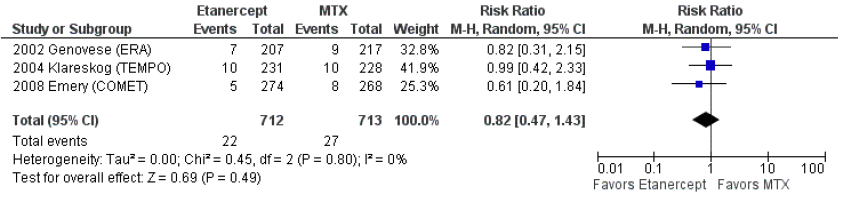


Figure 27. Tofacitinib 5 mg BID Risk Ratio (MTX-naive population)


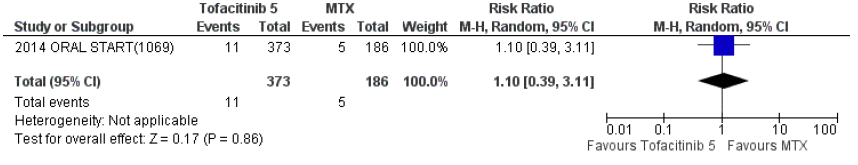


Figure 28. Tofacitinib 10 mg BID Risk Ratio (MTX-naive population)


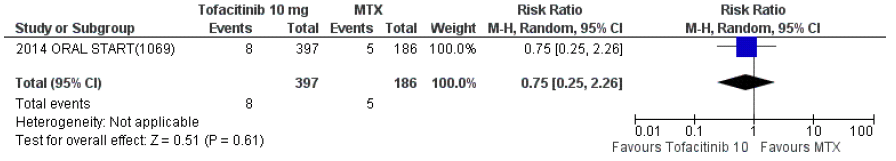


Figure 29. Tocilizumab Risk Ratio (MTX-naive population)


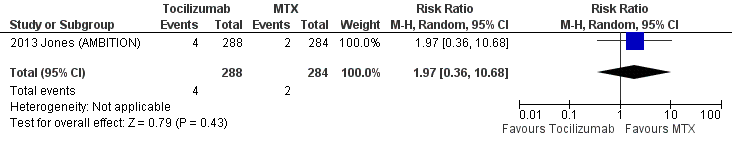


Figure 30. TNF Inhibitors Risk Ratio (MTX-naive population)


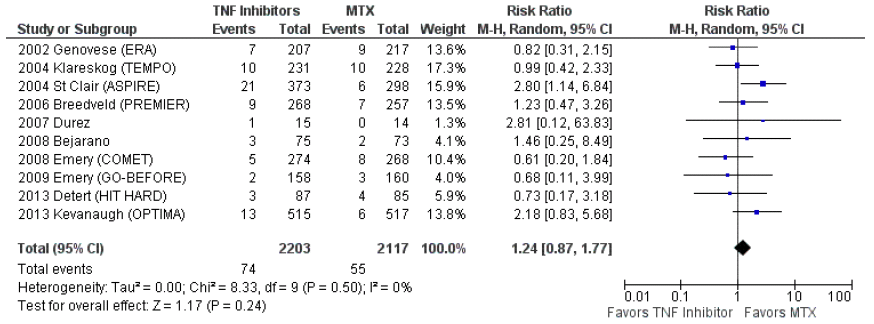


## Risk Difference by Drug

### DMARD-IR Population

Figure 31. Abatacept Risk Difference (DMARD-IR population)


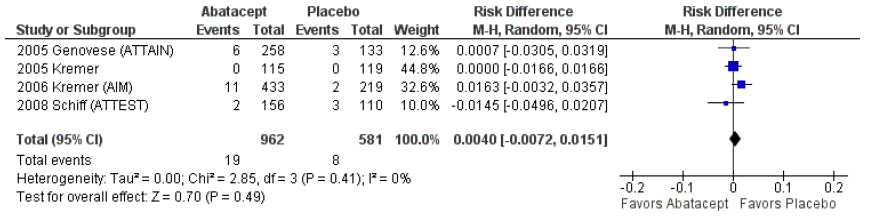


Figure 32. Rituximab Risk Difference (DMARD-IR population)


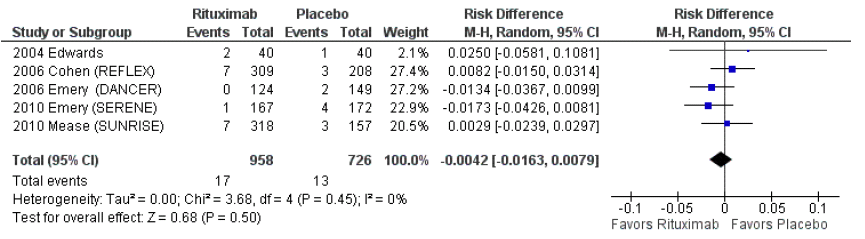


Figure 33. Tocilizumab Risk Difference (DMARD-IR population)


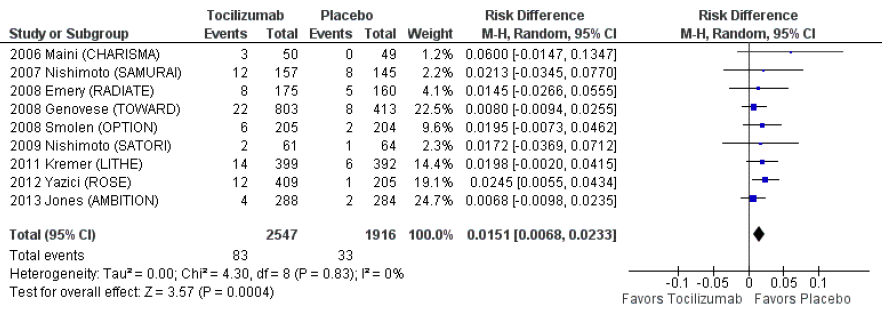


Figure 34. Infliximab Risk Difference (DMARD-IR population)


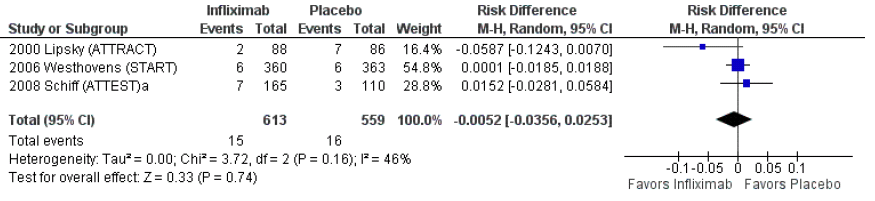


Figure 35. Golimumab Risk Difference (DMARD-IR population)


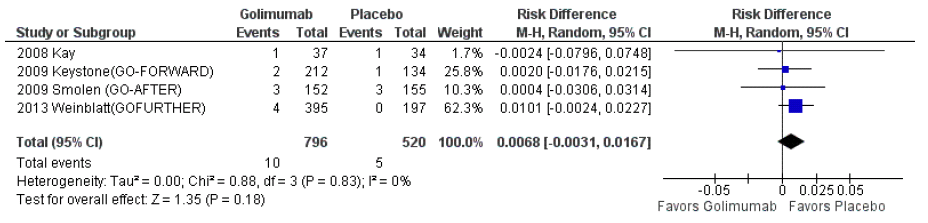


Figure 36. Certolizumab Risk Difference (DMARD-IR population)


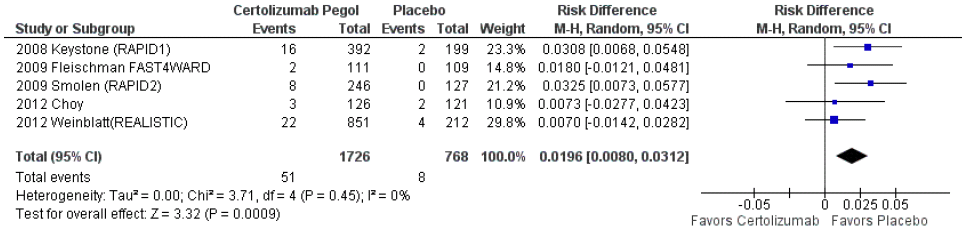


Figure 37. Adalimumab Risk Difference (DMARD-IR population)


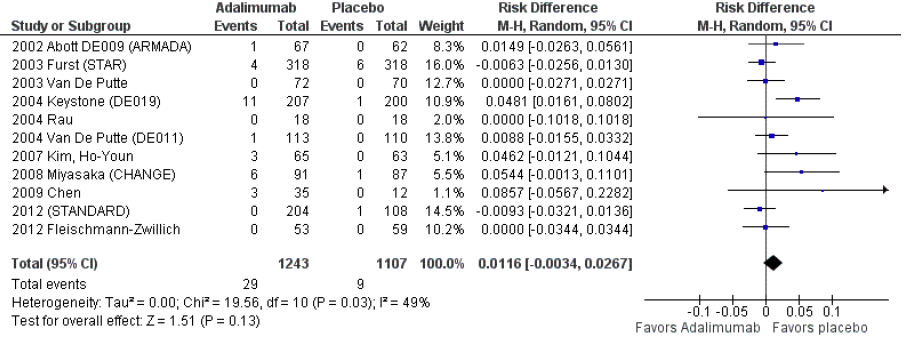


Figure 38. Etanercept Risk Difference (DMARD-IR population)


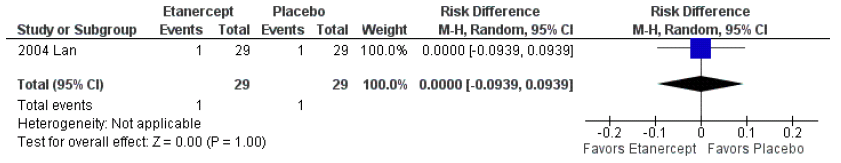


Figure 39. Tofacitinib 5 mg BID Risk Difference (DMARD-IR population)


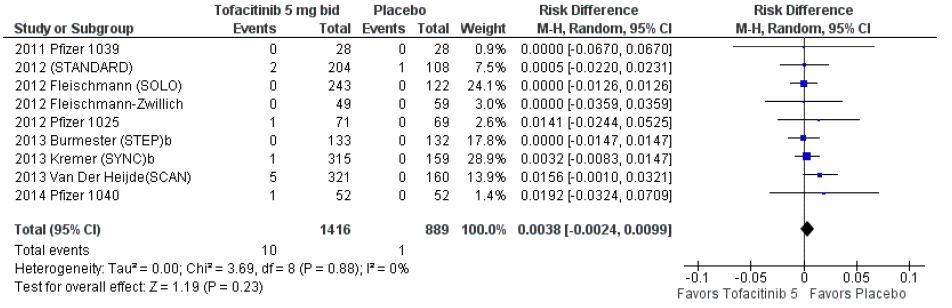


Figure 40. Tofacitinib 10 mg BID Risk Difference (DMARD-IR population)


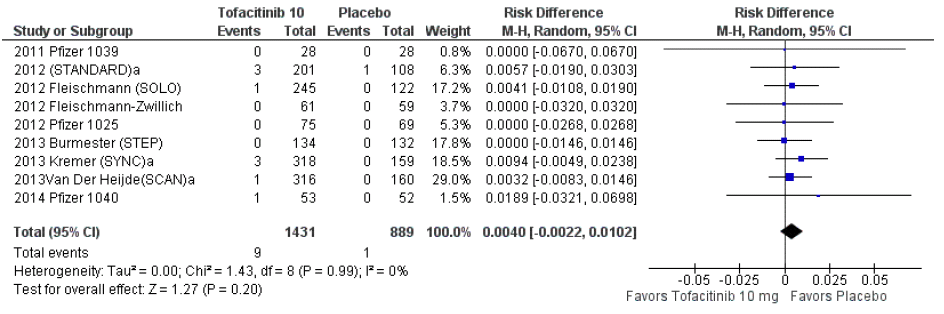


Figure 41. TNF Inhibitors Risk Difference (DMARD-IR population)


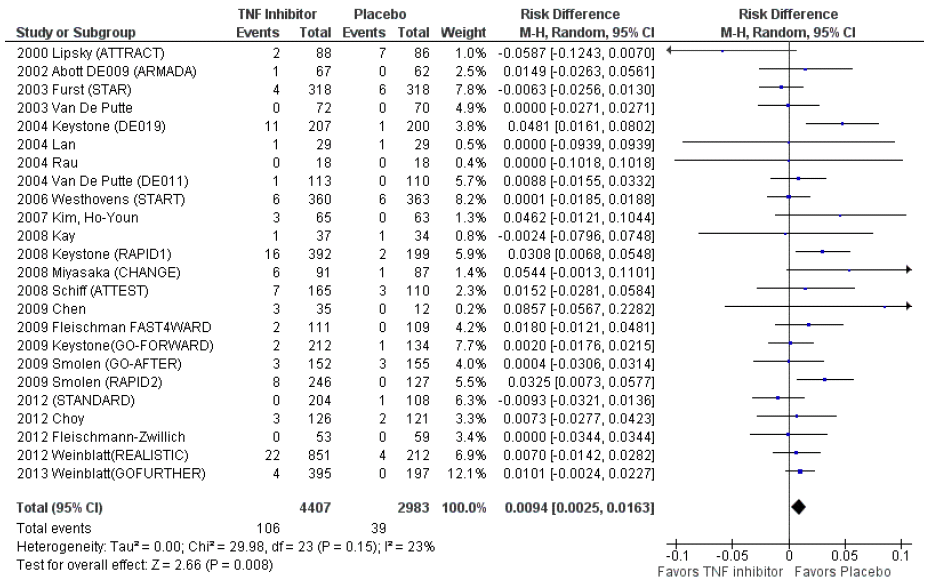


### MTX-naive Population

Figure 42. Abatacept Risk Difference (MTX-naive population)


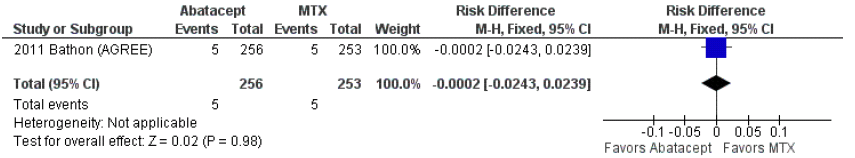


Figure 43. Rituximab Risk Difference (MTX-naive population)


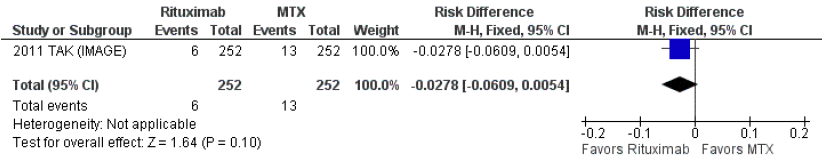


Figure 44. Infliximab Risk Difference (MTX-naive population)


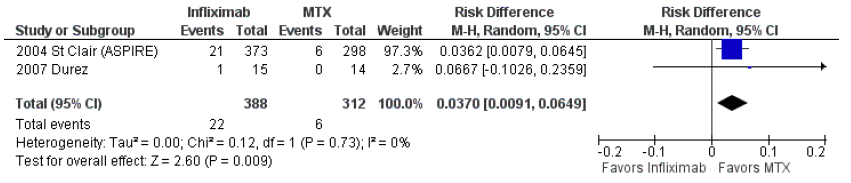


Figure 45. Golimumab Risk Difference (MTX-naive population)


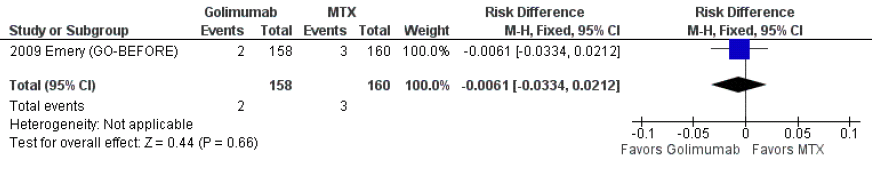


Figure 46. Adalimumab Risk Difference (MTX-naive population)


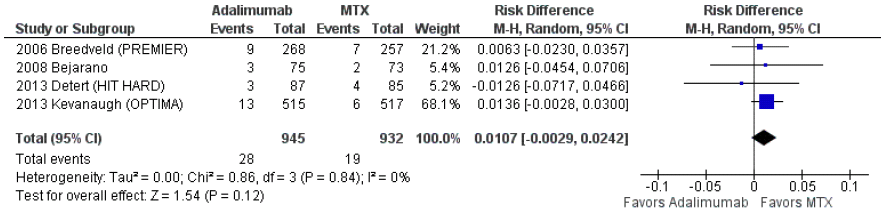


Figure 47. Etanercept Risk Difference (MTX-naive population)


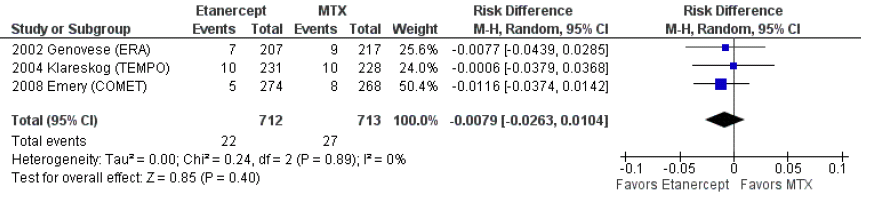


Figure 48. Tofacitinib 5 mg BID Risk Difference (MTX-naive population)


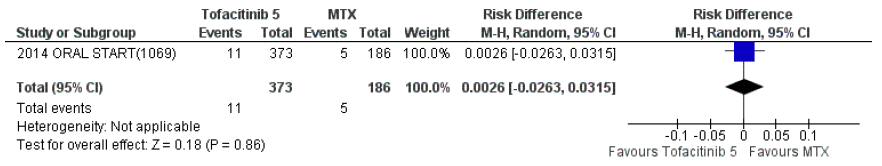


Figure 49. Tofacitinib 10 mg BID Risk Difference (MTX-naive population)


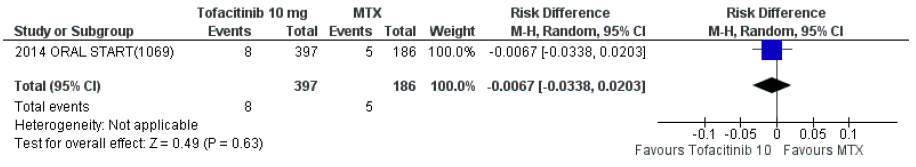


Figure 50. Tocilizumab Risk Difference (MTX-naive population)


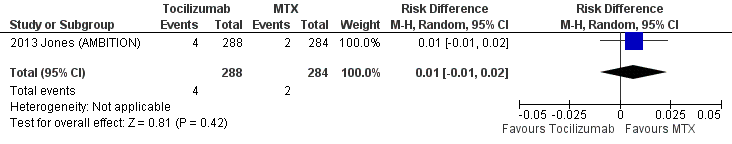


Date: 12-Nov-2014, Artifact ID 8250292

Figure 51. TNF Inhibitors Risk Difference (MTX-naive population)


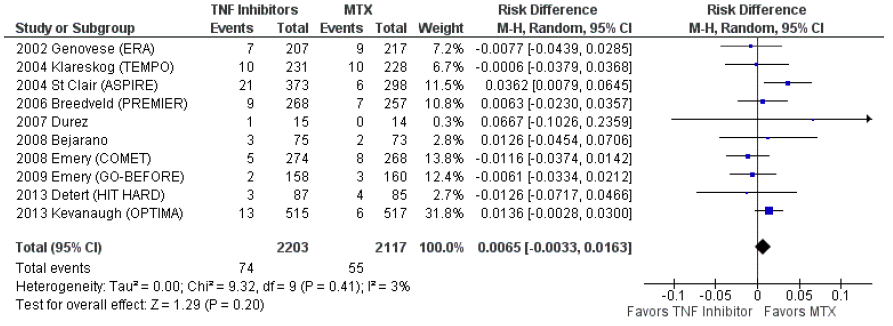


# FUNNEL PLOTS

Figure 52. Funnel plot for Abatacept Incidence Rate


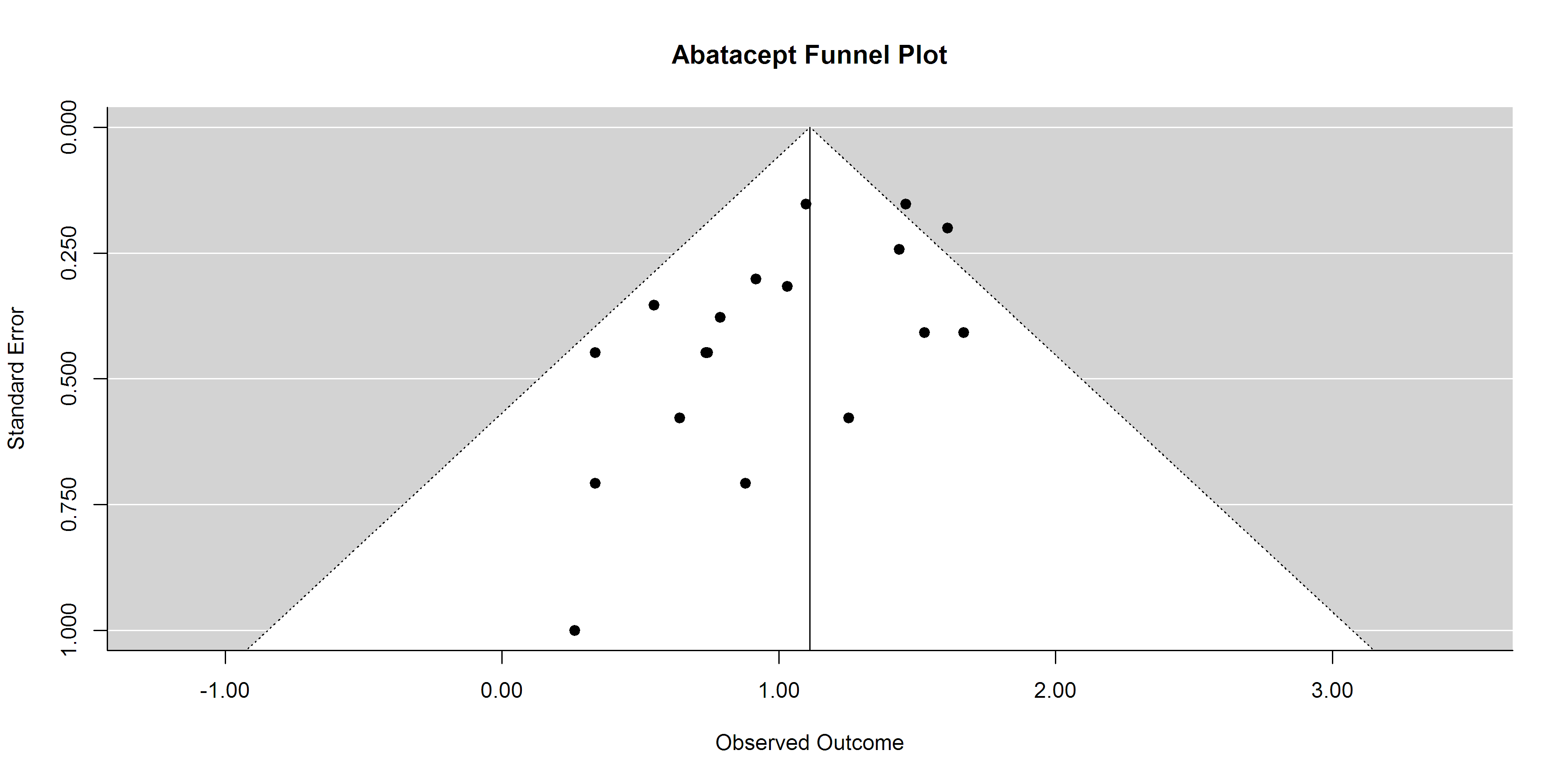


Figure 53. Funnel plot for Rituximab Incidence Rate


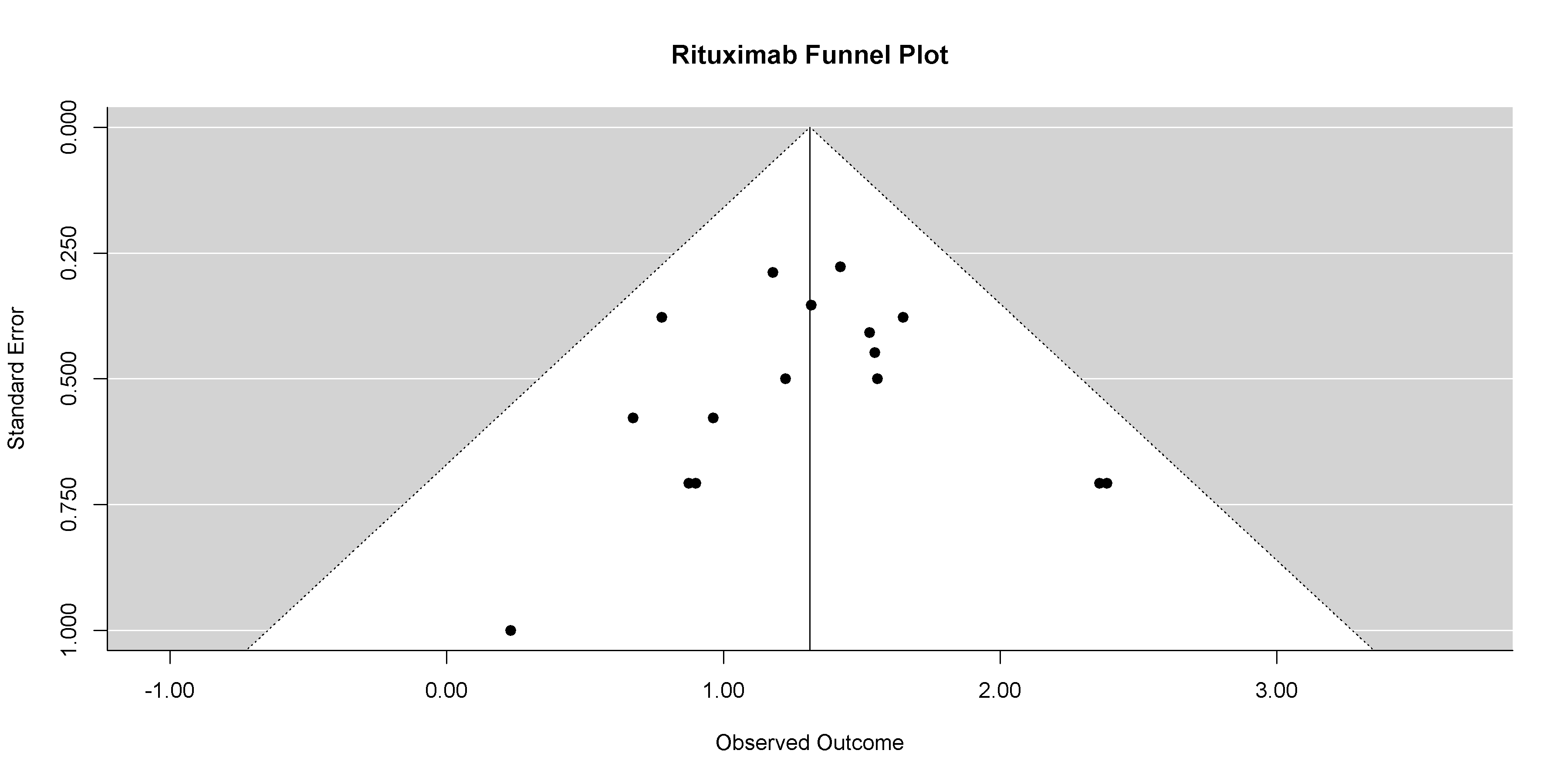


Figure 54. Funnel plot for Tocilizumab Incidence Rate


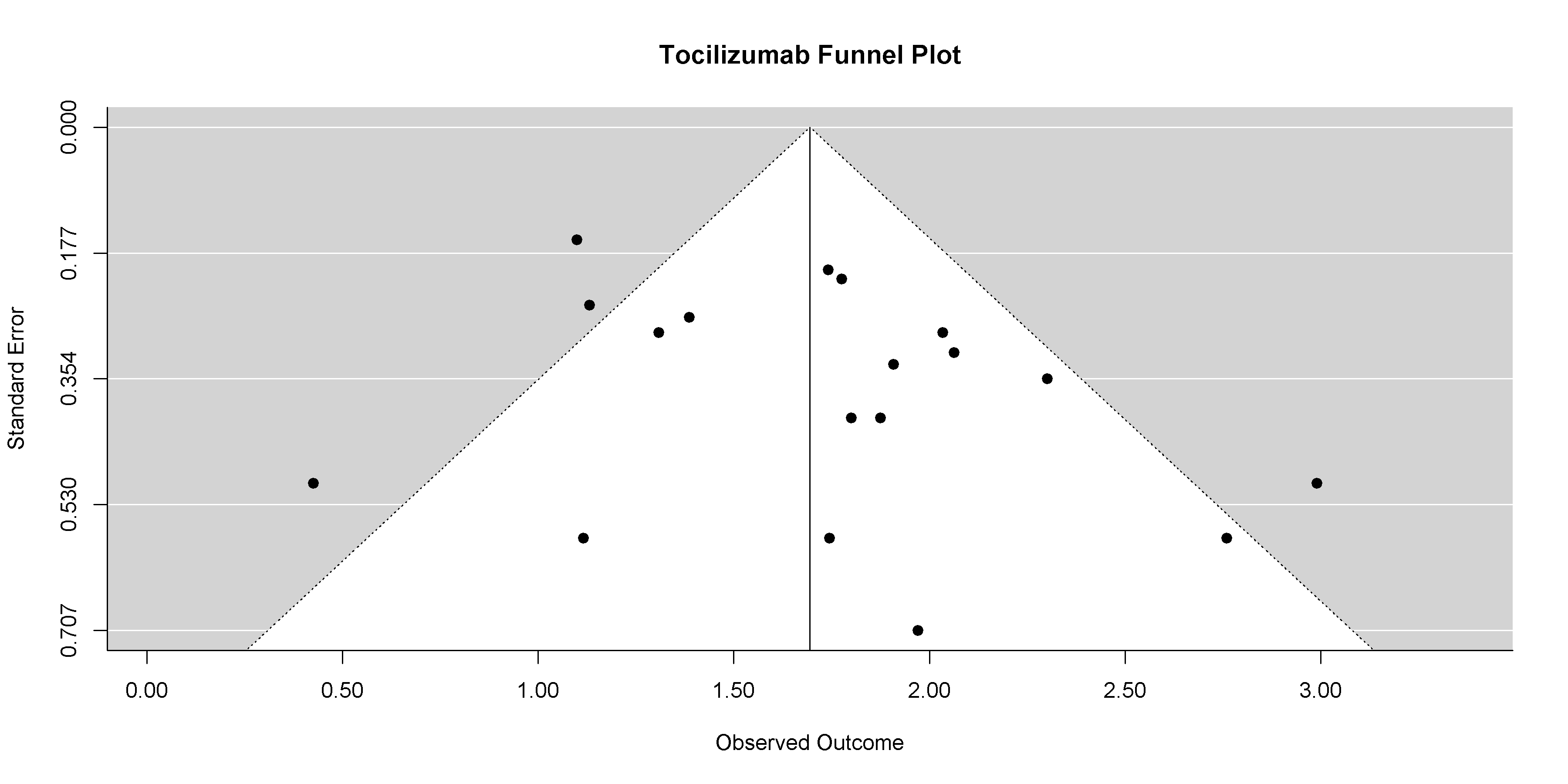


Figure 55. Funnel plot for Infliximab Incidence Rate


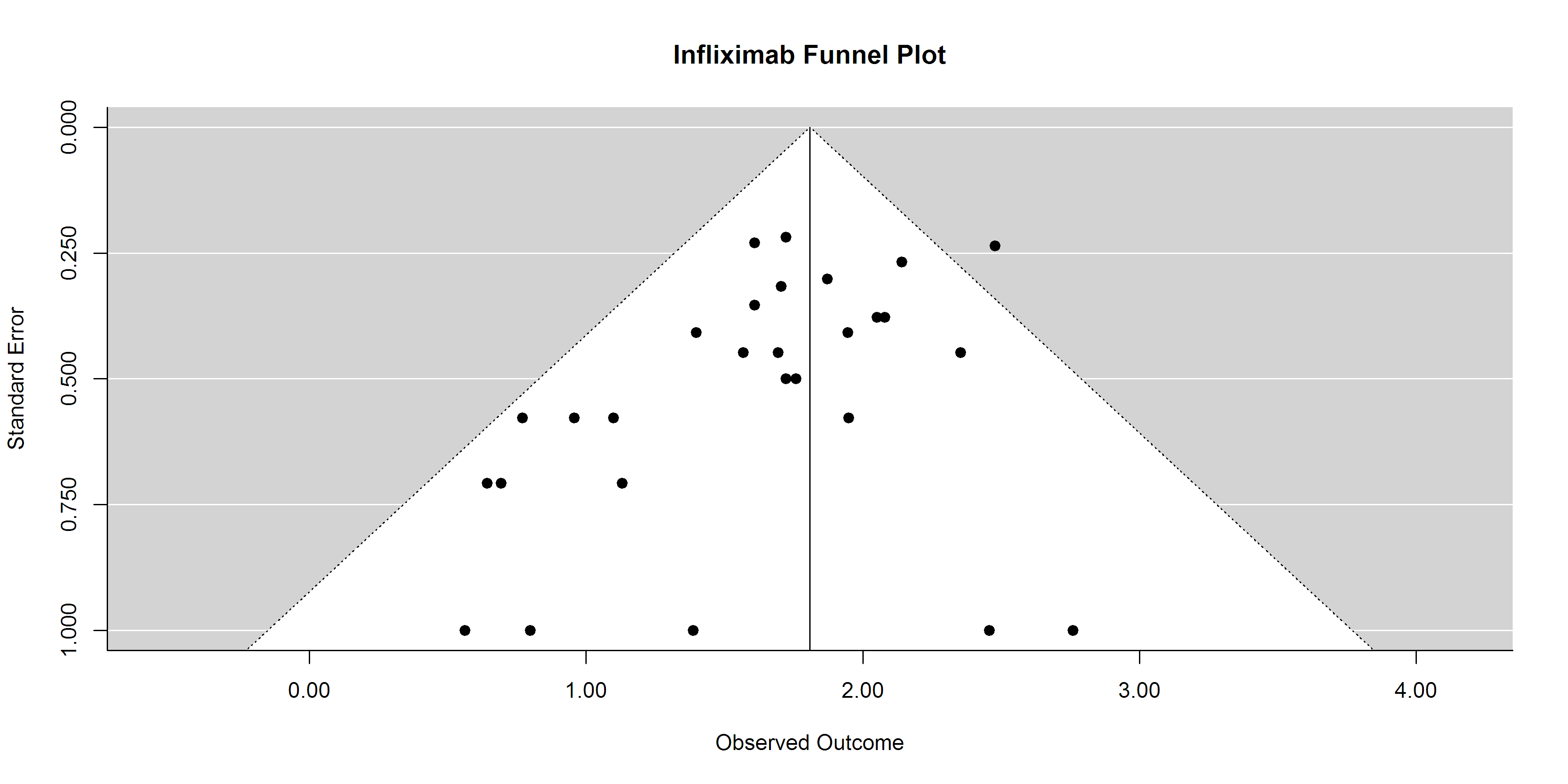


Figure 56. Funnel plot for Etanercept Incidence Rate


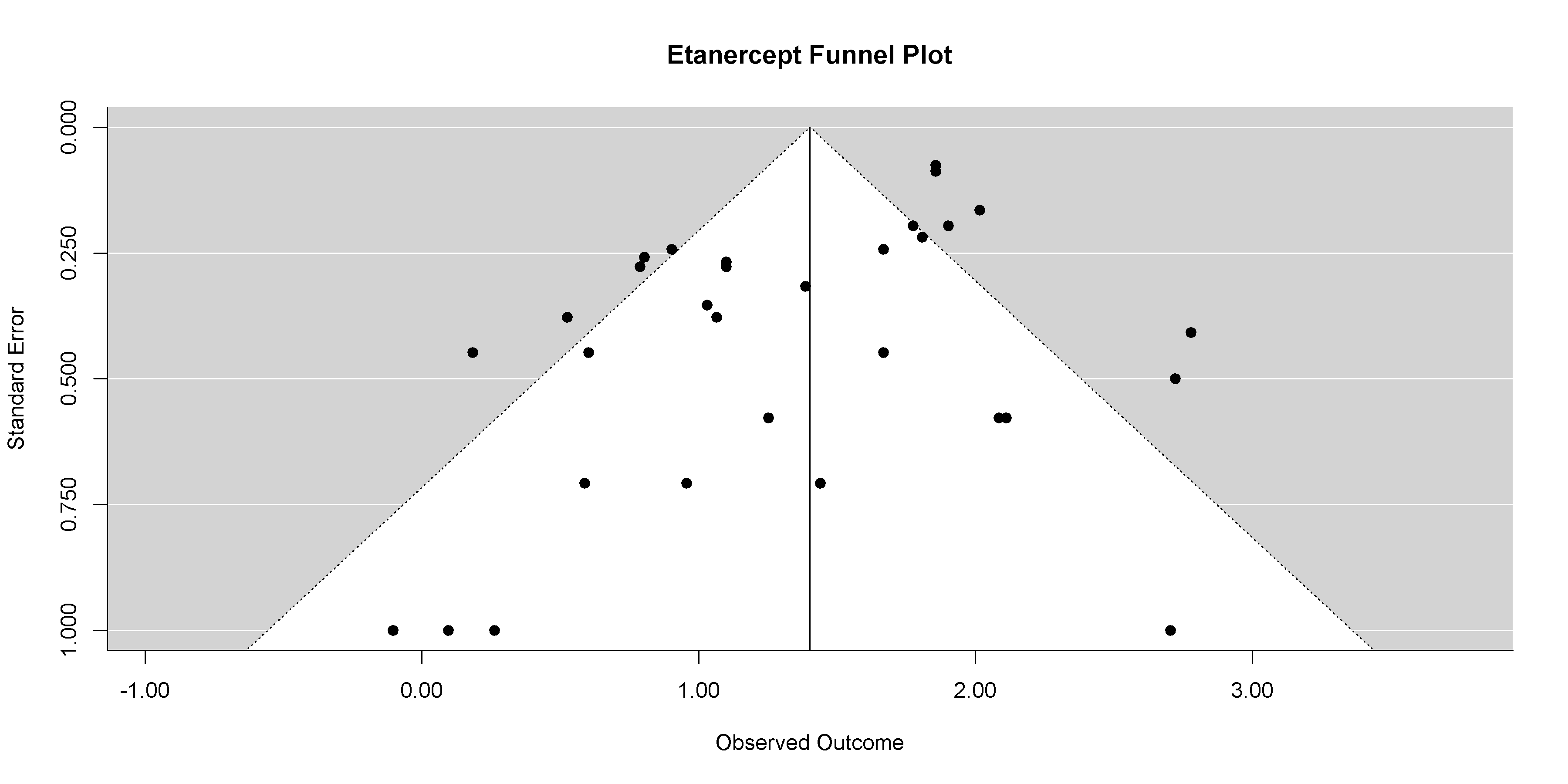


Figure 57. Funnel plot for Certolizumab Pegol Incidence Rate


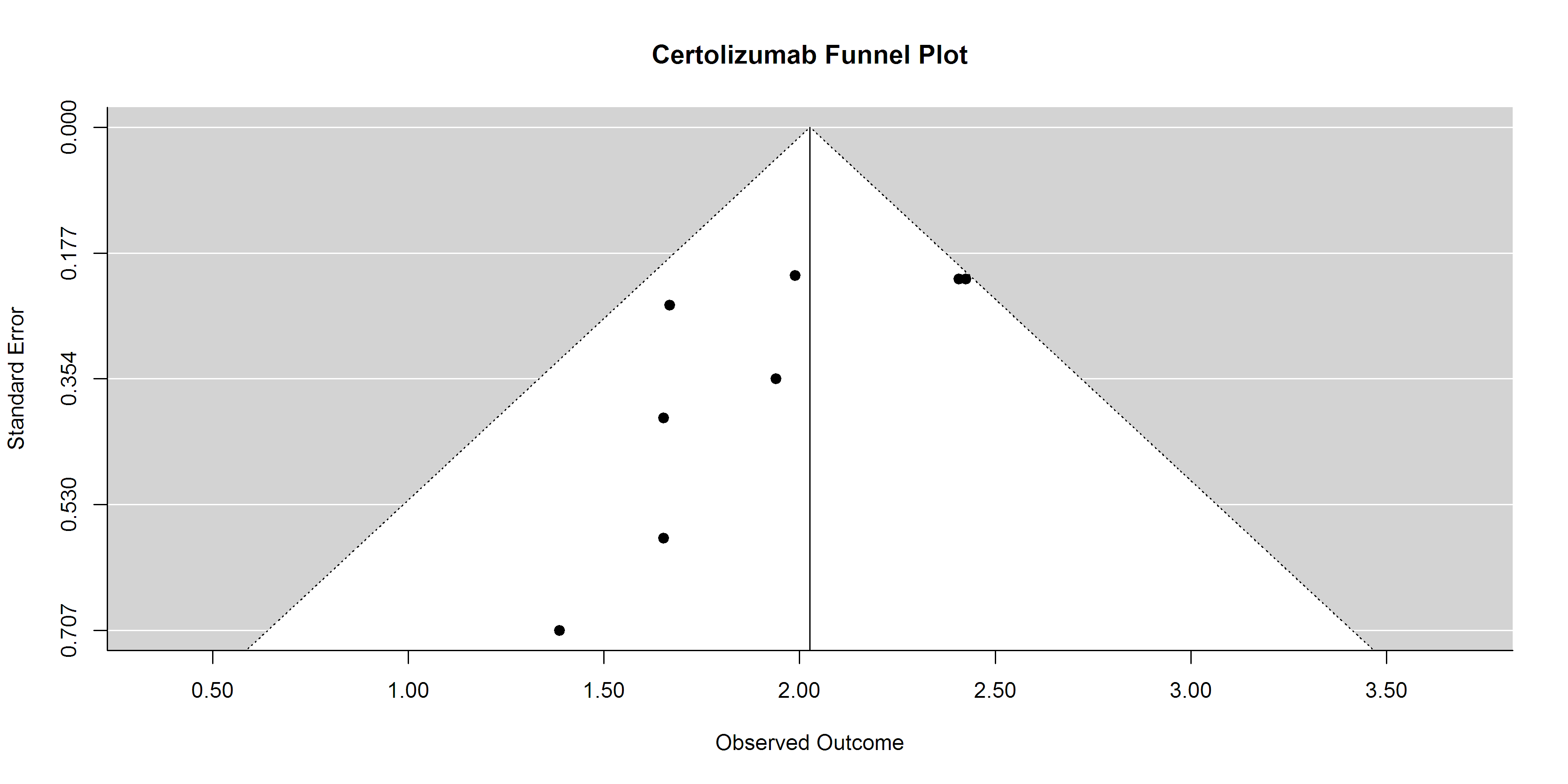


Figure 58. Funnel plot for Golimumab Incidence Rate


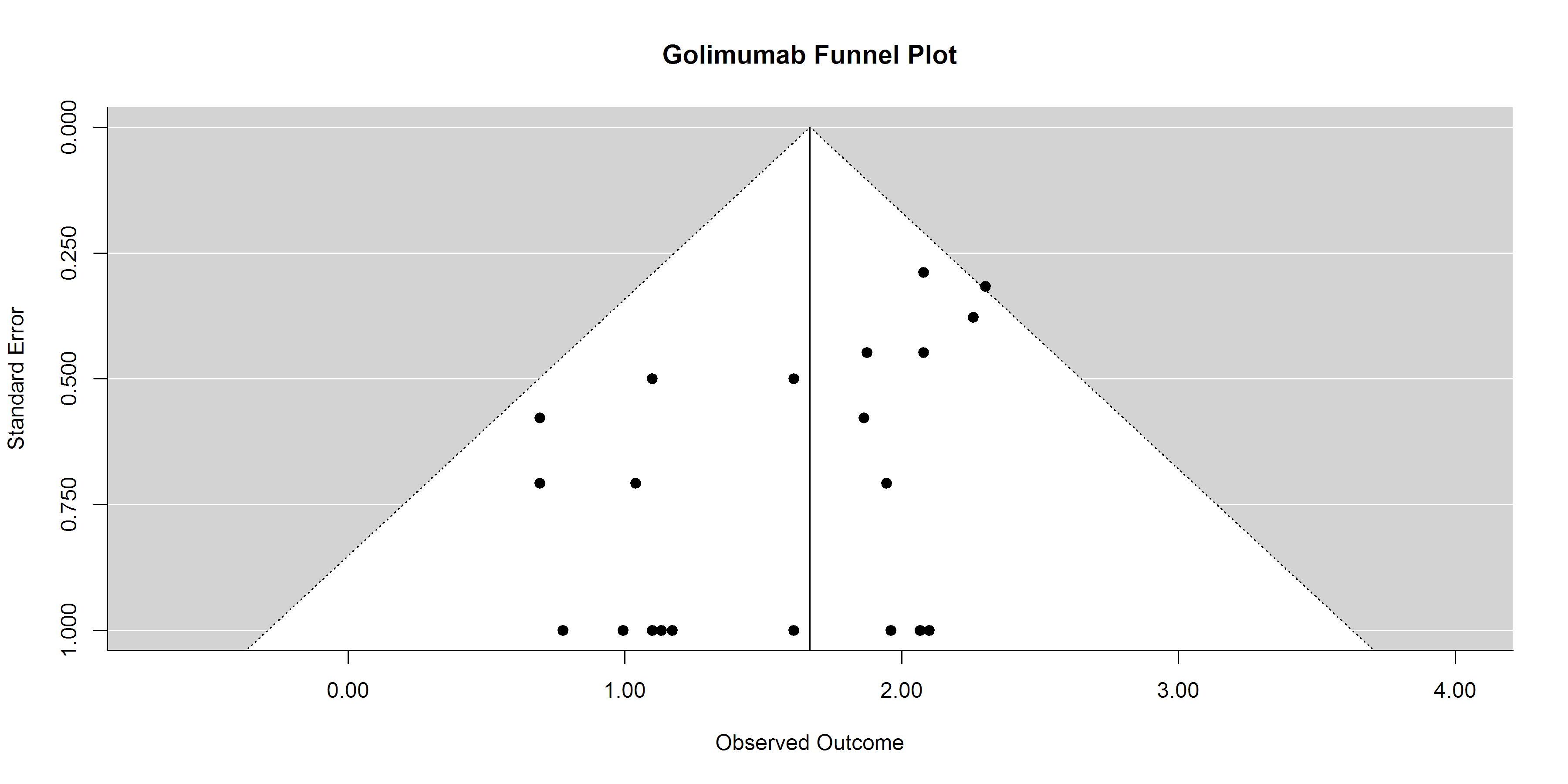


Figure 59. Funnel plot for Adalimumab Incidence Rate


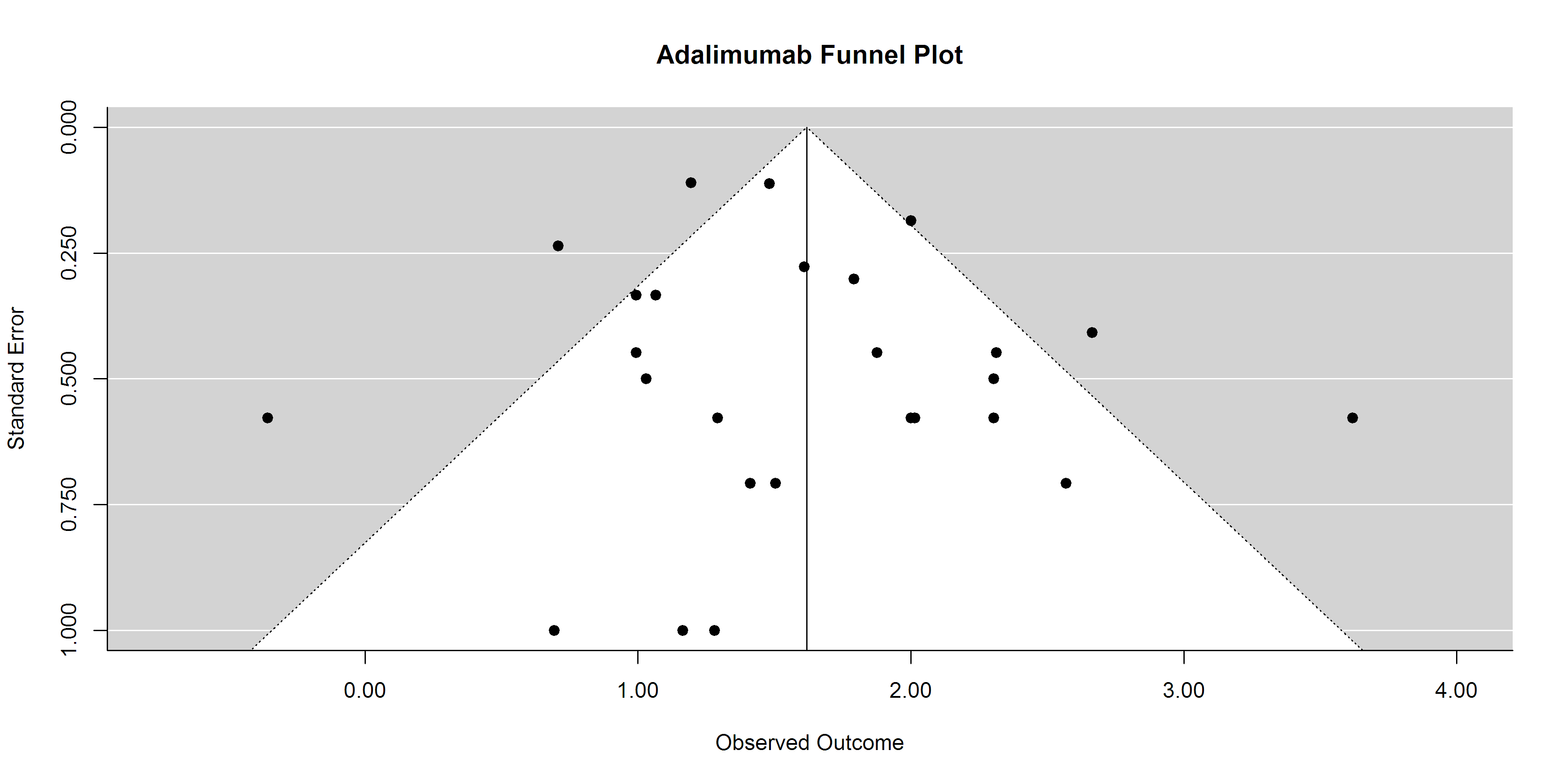


Figure 60. Funnel plot for TNF Inhibitors Incidence Rate


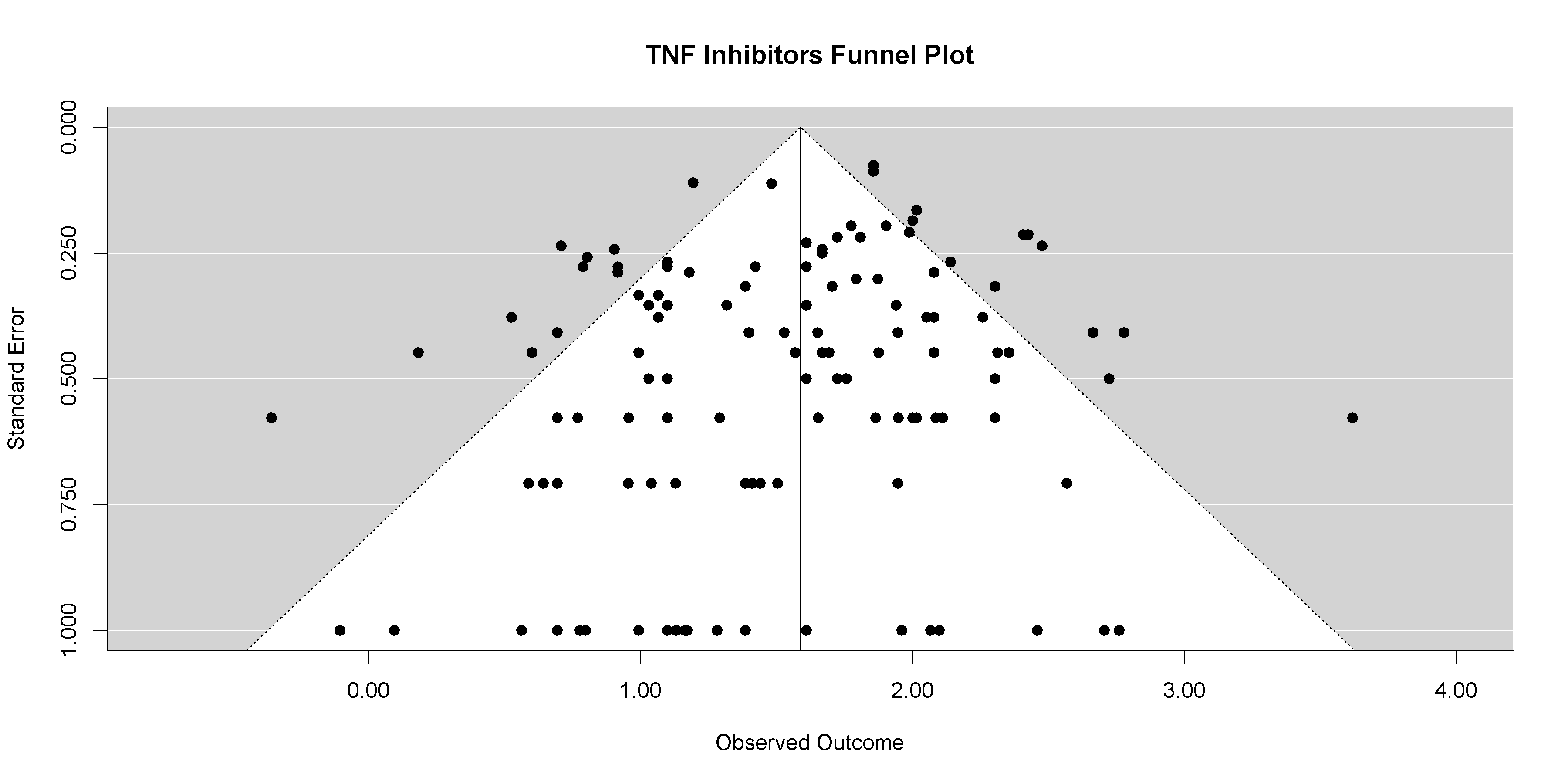


**SUPPLEMENTARY MATERIALS BIBLIOGRAPHY**
